# Computational Detection of Stage-Specific Transcription Factor Clusters during Heart Development

**DOI:** 10.3389/fgene.2016.00033

**Published:** 2016-03-23

**Authors:** Sebastian Zeidler, Cornelia Meckbach, Rebecca Tacke, Farah S. Raad, Angelica Roa, Shizuka Uchida, Wolfram-Hubertus Zimmermann, Edgar Wingender, Mehmet Gültas

**Affiliations:** ^1^University Medical Center Göttingen, Institute of Bioinformatics, Georg-August-University GöttingenGöttingen, Germany; ^2^Heart Research Center Göttingen, University Medical Center Göttingen, Institute of Pharmacology and Toxicology, Georg-August-University GöttingenGöttingen, Germany; ^3^DZHK (German Centre for Cardiovascular Research)Göttingen, Germany; ^4^Institute of Cardiovascular Regeneration, Goethe University FrankfurtFrankfurt, Germany; ^5^DZHK (German Centre for Cardiovascular Research)Frankfurt, Germany

**Keywords:** cardiomyogenesis, engineered heart muscle, MatrixCatch, Markov clustering, transcription factor collaboration

## Abstract

Transcription factors (TFs) regulate gene expression in living organisms. In higher organisms, TFs often interact in non-random combinations with each other to control gene transcription. Understanding the interactions is key to decipher mechanisms underlying tissue development. The aim of this study was to analyze co-occurring transcription factor binding sites (TFBSs) in a time series dataset from a new cell-culture model of human heart muscle development in order to identify common as well as specific co-occurring TFBS pairs in the promoter regions of regulated genes which can be essential to enhance cardiac tissue developmental processes. To this end, we separated available RNAseq dataset into five temporally defined groups: (i) mesoderm induction stage; (ii) early cardiac specification stage; (iii) late cardiac specification stage; (iv) early cardiac maturation stage; (v) late cardiac maturation stage, where each of these stages is characterized by unique differentially expressed genes (DEGs). To identify TFBS pairs for each stage, we applied the MatrixCatch algorithm, which is a successful method to deduce experimentally described TFBS pairs in the promoters of the DEGs. Although DEGs in each stage are distinct, our results show that the TFBS pair networks predicted by MatrixCatch for all stages are quite similar. Thus, we extend the results of MatrixCatch utilizing a Markov clustering algorithm (MCL) to perform network analysis. Using our extended approach, we are able to separate the TFBS pair networks in several clusters to highlight stage-specific co-occurences between TFBSs. Our approach has revealed clusters that are either common (NFAT or HMGIY clusters) or specific (SMAD or AP-1 clusters) for the individual stages. Several of these clusters are likely to play an important role during the cardiomyogenesis. Further, we have shown that the related TFs of TFBSs in the clusters indicate potential synergistic or antagonistic interactions to switch between different stages. Additionally, our results suggest that cardiomyogenesis follows the hourglass model which was already proven for *Arabidopsis* and some vertebrates. This investigation helps us to get a better understanding of how each stage of cardiomyogenesis is affected by different combination of TFs. Such knowledge may help to understand basic principles of stem cell differentiation into cardiomyocytes.

## 1. Introduction

Transcription factors (TFs) regulate the expression of genes and genetic programs to maintain survival and adaption to the environment in adult organisms as well as in embryo- and organogenesis. Most of them bind to recognized specific sequences in the DNA regulatory regions of genes and modify transcription, such as the assembly of the gene expression machinery. In mammalian tissues TFs often work in combinatorial interactions for precise regulation of specific programs (Boyer et al., 2005; Odom et al., 2006; Hu and Gallo, 2010; Neph et al., 2012). Such interactions can be positive, resulting in an enhanced expression of a gene or negative, resulting in reduced expression of a target gene. Thus, the identification of co-occurring transcription factor binding sites (TFBSs) in the promoter regions of regulated genes indicate potential combinatorial interactions between TFs that are important for understanding the molecular mechanisms, e.g., of tissue development during embryogenesis.

The human heart is the first organ formed during embryogenesis (Kirby, 2002; Brand, 2003; Buckingham et al., 2005; Brewer and Pizzey, 2006; Schleich et al., 2013), and it consists of different cell types, which develop simultaneously and are regulated by TFs as well as their combinatorial interactions. Until now, several groups analyzed TFs and their influence on cardiac development (Ryan and Chin, 2003; Pikkarainen et al., 2004; Peterkin et al., 2005; Brewer and Pizzey, 2006; Martin et al., 2010; Shi and Jin, 2010; Turbendian et al., 2013; Chaudhry et al., 2014; Takeuchi, 2014; Wang and Jauch, 2014). These studies mainly focus on individual TFs or their related families e.g., GATA family, TBX family, or NKX2 family (Ryan and Chin, 2003; Pikkarainen et al., 2004; Miura and Yelon, 2013; Turbendian et al., 2013). However, a detailed analysis of interactions between TFs and their role in cardiac development is limited to interactions between known cardiac TFs like NKX2-5 or MEF2 which are essential for the generation of cardiac tissues from stem cells (Martin et al., 2010; Sylva et al., 2014; Takeuchi, 2014). A complete survey of potential TF interactions by co-occurring TFBSs in the promoter regions of genes which regulate cardiac development is still missing, but needed to understand embryonic cardiac development, in particular of cardiomyocytes (CMs).

CMs comprise the most important functional cells in the human heart (Ye et al., 2013; Sylva et al., 2014). CMs show a limited potential to regenerate after myocardial infarction or other cardiovascular diseases (CVDs), which is at maximum 50% CM renewal per lifetime and less than 1% per year (Bergmann et al., 2009; Sylva et al., 2014; Takeuchi, 2014). Replacing CMs in elderly by for example enhanced cardiomyocyte proliferation may improve the quality of their life, but requires an understanding of how CMs develop and of how they can be replaced (Akhurst, 2012; Ye et al., 2013; Euler, 2015).

One approach is to apply tissue engineered myocardium to restore muscle mass and thus reintroduce contractility (Zimmermann et al., 2006). Such tissues can be generated from embryonic stem cells (ESCs), induced pluripotent stem cells (iPSCs), or parthenogenetic stem cells (Soong et al., 2012; Didié et al., 2013; Ye et al., 2013; Tiburcy and Zimmermann, 2014). Controlling cardiomyogenesis *in vitro* requires insight into biological processes governing embryonic heart development. To understand cardiac development from a systems biology perspective, identification of the mechanisms controlling the expression of fate determining TFs and their regulation of transcription are of fundamental importance. Co-occurring TFBSs in the regulatory regions of genes which are specific for a particular developmental stage reveal potential TF interactions that are likely to regulate these stages. There are in fact plenty of TF-TF interactions known as implicated in organogenesis, but the specific time points when particular interactions occur, are difficult to obtain and mostly not annotated in public databases. Only intense literature surveys provide such information.

Recent studies identifying the co-occurrence of TF pairs focus either on combinatorial approaches where e.g., specific DNA-sequences bound by different TFs simultaneously were selected from a library of random sequences (Jolma et al., 2015) or approaches that focus on data integration e.g., ChIP-seq, SELEX together with Hi-C to reveal long-range chromatin interactions (Jolma et al., 2013; Wong et al., 2016). Although the selection of interacting TF pairs from a library of random sequences underpins potential interactions of TFs, it does not give any hints on the actual interactions in particular cell types or tissues. Data integration and especially Hi-C technology is very promising for the future, but currently there is a lack in publicly available data sets that cover the time dependent organogenesis of the human heart.

In this study we analyze a time series dataset obtained from RNAseq at different time points of in vitro cardiomyogenesis (Hudson et al.; in revision) to identify co-occurring TFBSs which indicate potential interacting TFs that are crucial for understanding the gene regulatory mechanisms during the heart development. The dataset consists of six different time points (day: 0, 3, 8, 13, 29, and 60) where the gene expression in the tissue culture was measured by RNAseq. The data comprises early heart development in general and can be differentiated in the following major developmental stages: (i) mesoderm induction stage (day 0–day 3); (ii) cardiac specification stage (day 3–day 13; early 3–8, late 8–13); (iii) cardiac maturation stage (day 13–day 60; early 13–29, late 29–60). For each stage we determined the set of unique differentially expressed genes (DEGs) utilizing *limma* on the FPKM-values in the dataset (Smyth, 2004). To identify specific TF interactions in individual stages, we analyzed the promoter sequences of corresponding DEGs employing the MatrixCatch approach (Deyneko et al., 2013). As a result, we observed a set of co-occurring TFBSs for each stage whose corresponding TFs are likely to represent potential core regulators of a particular developmental stage. Although the analyzed DEGs are unique in each stage, the identified TFBS pairs are highly overlapping between stages. To overcome this problem in MatrixCatch results, we further applied Markov clustering algorithm (MCL; Dongen, 2000) for the detection of clusters which contain stage specific co-occurrences between TFBSs. In recent years, MCL has gained great attention in the bioinformatics community for the detection of high-quality clusters in biological networks due to its highly effective and successful algorithm. Especially, for the clustering of protein-protein interaction networks, several studies have shown that MCL is superior to conventional clustering approaches in terms of detection of high-quality and more accurate functional clusters (Brohée and van Helden, 2006; Vlasblom and Wodak, 2009; Shih and Parthasarathy, 2012). These articles encouraged us to utilize MCL for the elimination of negligible pairs at each stage and thus for the determination of remaining TFBS pairs, which may play crucial roles during cardiomyogenesis. To this end, we focused on clusters whose central binding site is present at almost all stages, but its partners differ stage-specifically. These clusters may regulate DEGs in each stage and are likely to be fundamentally implicated in cardiac muscle development.

## 2. Materials and methods

**Figure 1:**
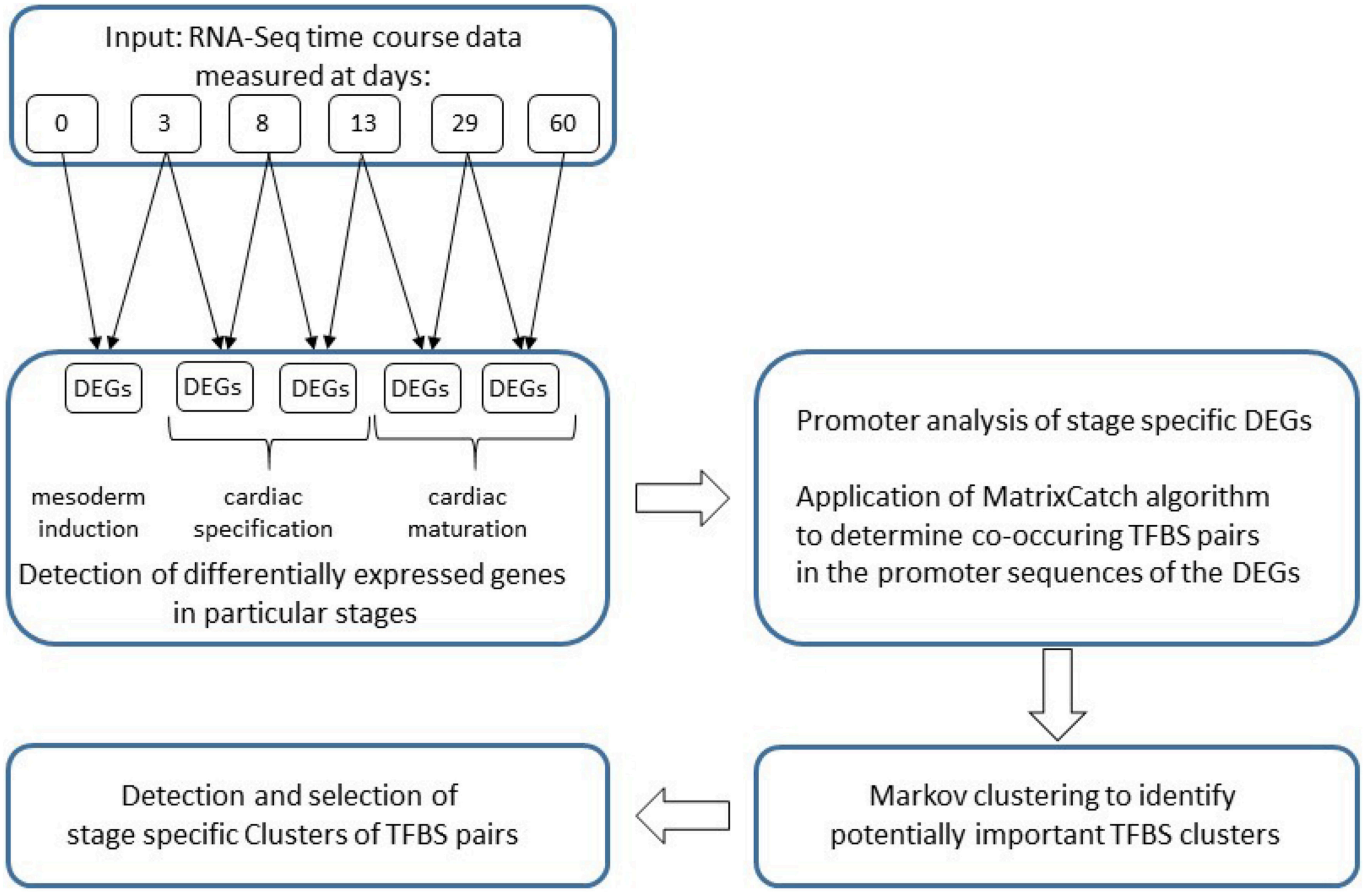
**Flowchart of the analysis applied in this study**.

In this section we describe the differentially expressed genes analyzed and the methods applied and partly developed. Our analysis follows the structure of Figure 1.

### 2.1. Selection of differentially expressed genes

The data, available as a FPKM normalized RNAseq time series, was mapped to corresponding gene symbols (hgnc-symbols) and further analyzed using *limma* package from the Bioconductor project for R with standard procedures (Smyth, 2004; R Core Team, 2015). The time series data describe human cardiomyogenesis in vitro at time points day 0, 3, 8, 13, 29, and 60, whereas day 0 resembles blastocyst stage development and day 60 early fetal stages (Hudson et al.; in revision). We calculated DEGs between two time points which define a particular developmental stage where: (i) day 0–3 defines the mesoderm induction stage; (ii) day 3–8 early cardiac specification; (iii) day 8–13 late cardiac specification; (iv) day 13–29 early cardiac maturation and; (v) day 29–60 the late cardiac maturation stage (this stage describes the transition from an embryonic to a fetal cardiac maturation stage). We filtered the set of all DEGs for protein coding genes (excluding TFs) and their uniqueness in a stage by comparison to all other stages with *p*-value ≤ 0.05 and FDR ≤ 0.01 (see Supplementary File 1). A heatmap of stage-specific DEGs is given in Supplementary File 2.

### 2.2. Promoter sequences

Using UCSC genome browser (Karolchik et al., 2004), we extracted for each protein coding gene (RefSeq gene) based on its annotated transcription start site (TSS) the -1 kb putative regulatory promoter region.

It is important to note that, according to TSS annotations, a RefSeq gene can have multiple overlapping promoter regions which results in overestimation of the importance of some transcription factor binding sites (TFBSs). Thus, following the line of PC-TraFF to remove the redundancy between sequences, we filtered them regarding their TSSs (Meckbach et al., 2015). Consequently, we used in our analysis only those sequences which have no overlap.

In this study, the assembly of the hg19 release of the human genome was used and only UCSC track refGene annotations were considered which correspond to the chromosomes chr1-chr22, chrX, and chrY.

### 2.3. MatrixCatch analysis

MatrixCatch is a novel method introduced by Deyneko et al. (2013) to recognize experimentally verified TF pairs based on the co-localization of their TFBSs, known as composite regulatory modulues (CRMs), in single promoters. To detect CRMs in the individual sequences under study, MatrixCatch scans each sequence and its reverse complement using a special library of position weight matrices (PWMs). This library has been specified by considering the TF binding scores, relative orientations and distances between TFs that are experimentally known to interact, as documented in the TRANSCompel database (Kel-Margoulis et al., 2002). Consequently, the usage of MatrixCatch yields an important practical advantage since this method provides a high number of known CRMs in sequences with their biological interpretation (for details, see Deyneko et al., 2013).

In our study, we applied MatrixCatch to the promoter sequences of the filtered DEGs of the different heart developmental stages. As we have recently suggested in PC-TraFF (Meckbach et al., 2015), we prefer in this study the usage of TFBS pairs instead of CRMs, since those pairs were detected in a set of sequences. This indicates the importance of potential collaborations between corresponding TFs in the gene set of interest.

### 2.4. Clustering of co-occurring TFBSs

Since MatrixCatch provides all detected TFBS pairs of experimentally verified TF interactions in promoters, the detected pairs are highly overlapping between developmental stages. To differentiate stage specific roles of TFBS pairs, we first determined the frequency of each pair in MatrixCatch results. After that, we applied the Markov clustering algorithm (MCL; Dongen, 2000) which is able to eliminate negligible TFBS pairs based on their frequencies at each stage. To this end, we constructed an interaction network based on the TFBS pairs for each heart developmental stage, where nodes are TFBSs and edges display the co-occurrences between them.

Let N: = (V, E) be an undirected interaction network of TFBS pairs where any two elements (*v*_*i*_, *v*_*j*_ ∈ V) of N are connected by an edge *e*_(*v*_*i*_, *v*_*j*_)_ belonging to E, if and only if the corresponding TFBS pair was identified by MatrixCatch. Further, *w*(*v*_*i*_, *v*_*j*_) denotes the weight of an edge *e*_(*v*_*i*_, *v*_*j*_)_, which represents the observed frequency of the TFBS pair (*v*_*i*_, *v*_*j*_) found by MatrixCatch in the promoter sequences of genes under study.

Based on the weights of edges, an adjacency matrix A_*n*×*n*_ of each network was constructed as

$$\begin{array}{l} A_{i,j}=\{\begin{array}{ll} w(v_{i},v_{j}) & \text{ if }e_{\left(v_{i},v_{j}\right)}\in E\;\\ 0 & \text{ else}. \end{array} \end{array}$$

A_*n*×*n*_ was then converted into a row stochastic “Markov” matrix M_*n*×*n*_, where *m*_*i*×*j*_ represents the transition probability between nodes *v*_*i*_ and *v*_*j*_ in the network under study. The most common way to construct a row stochastic transition matrix M is the normalization of rows in A to sum to 1. This process can be simply given as: M = Δ^−1^·A, where Δ is a *n*×*n* diagonal degree matrix and defined as:

$$\begin{array}{l} \Delta =\left(\begin{array}{cccc} d_{1} & 0 & \cdots & 0\\ 0 & d_{2} & \cdots & 0\\ \vdots & \vdots & \ddots & 0\\ 0 & 0 & \cdots & d_{n} \end{array}\right)=\left(\begin{array}{cccc} \sum_{j=1}^{n}a_{1j} & 0 & \cdots & 0\\ 0 & \sum_{j=1}^{n}a_{2j} & \cdots & 0\\ 0 & 0 & \ddots & 0\\ 0 & 0 & \cdots & \sum_{j=1}^{n}a_{nj} \end{array}\right) \end{array}$$

Based on matrix M, we employed MCL (Dongen, 2000) to detect densely connected TFBSs in each network. Briefly, the basic intuition of MCL was based on a simulation of stochastic flows on the underlying interaction network to separate high-flow regions from low-flow regions. To this end, Expand and Inflate operations were applied on M until M reaches its steady state. While the Expand operation corresponds to matrix multiplication (M = M×M), the Inflate operation is used to increase the contrast between higher and lower probability transitions by taking each entry *m*_*i*×*j*_ in M to the power of inflation parameter *r* > 1. Finally, M was re-normalized into a row stochastic matrix. The pseudo-code for MCL is given in Algorithm 1.

**Algorithm 1:** Markov Clustering Algorithm

**Input:** M and *r* > 1
**Output:** C: A list of clusters
**Methode:**
1: *t* = 0
2: M_*t*_ = M
3: **repeat**
4: *t* = *t* + 1
5: M_*t*_ = *Expand*(M_*t*−1_) = M_*t*−1_×M_*t*−1_
6: $M_{t}=Inflate\left(M_{t},r\right)={\left\{\frac{{\left(m_{ij}\right)}^{r}}{\overset{n}{\underset{k=1}{\sum }}{\left(m_{ik}\right)}^{r}}\right\}}_{i,j=1}^{n}$
7: **until** M_*t*_ converges
8: C: clusters(M_*t*_)

## 3. Results

We analyzed a time course data set which covers heart muscle development in human embryonic stem cell derived tissue cultures at days 0, 3, 8, 13, 29, and 60 (Hudson et al., in revision). These time points cover the mesoderm induction stage (day 0–day 3), the cardiac specification stage (day 3–day 13), and the cardiac maturation stage (day 13–day 29). We further defined cardiac specification and cardiac maturation into two more stages, i.e.,: (i) early cardiac specification and maturation stage from days 3–8 and days 13–29, respectively; (ii) late cardiac specification and maturation with transition from embryonic to fetal stages defined by culture days 8–13 and days 29–60, respectively. By comparison of neighboring time points, for each stage, we determined the set of DEGs and filtered them according to their uniqueness in a particular stage. Afterwards, we utilized MatrixCatch to identify co-occurring pairs of TFBSs in the promoter regions of these DEGs. Consequently, we identified: (i) 63 TFBS pairs based on 429 DEGs for the mesoderm induction stage; (ii) 82 TFBS pairs based on 1233 DEGs for the early cardiac specification stage; (iii) 24 TFBS pairs based on 36 DEGs for the late cardiac specification stage; (iv) 52 TFBS pairs based on 205 DEGs for the early cardiac maturation stage; (v) 76 TFBS pairs based on 964 DEGs for the late cardiac maturation stage (see Supplementary File 3).

Due to underlying methodology of MatrixCatch, the detected TFBS pairs show a large overlap between different stages although they may play different roles in these stages. To reduce this drawback of MatrixCatch, we further applied Markov clustering algorithm that seeks to remove negligible TFBS pairs by emphasizing the roles of remaining pairs at each stage. Consequently, we obtained (i) 19 clusters for the mesoderm induction stage; (ii) 25 clusters for the early cardiac specification stage; (iii) 11 clusters for the late cardiac specification stage; (iv) 21 clusters for the early cardiac maturation stage, and (v) 24 clusters for the late cardiac maturation stage (see Supplementary File 4).

**Figure 2:**
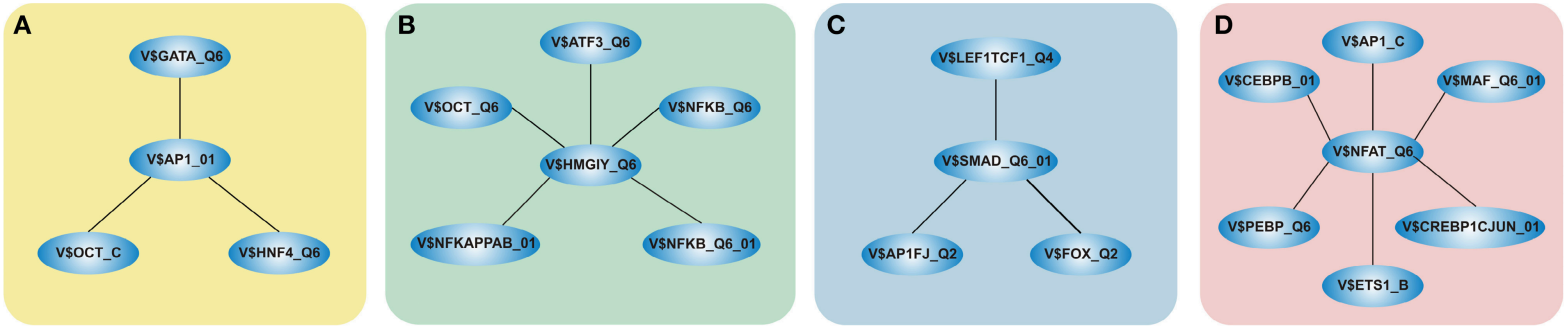
**Clusters we focus on in our analysis in the order in which they are analyzed in this study**. The clusters comprise all interactions during the complete time course, identified by employing MatrixCatch and MCL. The constitution of each cluster for a particular stage is shown in the corresponding tables. **(A)** shows the AP-1-cluster, Table 1; **(B)** HMGIY-cluster, Table 2; **(C)** SMAD-cluster, Table 4; **(D)** NFAT-cluster, Table 5.

We focused only on clusters with V$AP1_01, V$HMGIY_Q6, V$SMAD_Q6_01, and V$NFAT_Q6 binding sites in their center (see Figure 2), because these clusters contain at least three interactions and the changes in their constitution provide crucial information about different cardiac developmental stages. We analyzed the TFBS pairs in these clusters according to their potential role in cardiac development. We omitted clusters, when the expression values of TF genes are below a certain threshold or their importance in heart development is currently unknown. For our analysis, we applied a FPKM threshold value of 10, which discriminates robustly between expressed TF genes and low or not expressed TF genes.

### 3.1. AP-1-cluster

**Figure 3:**
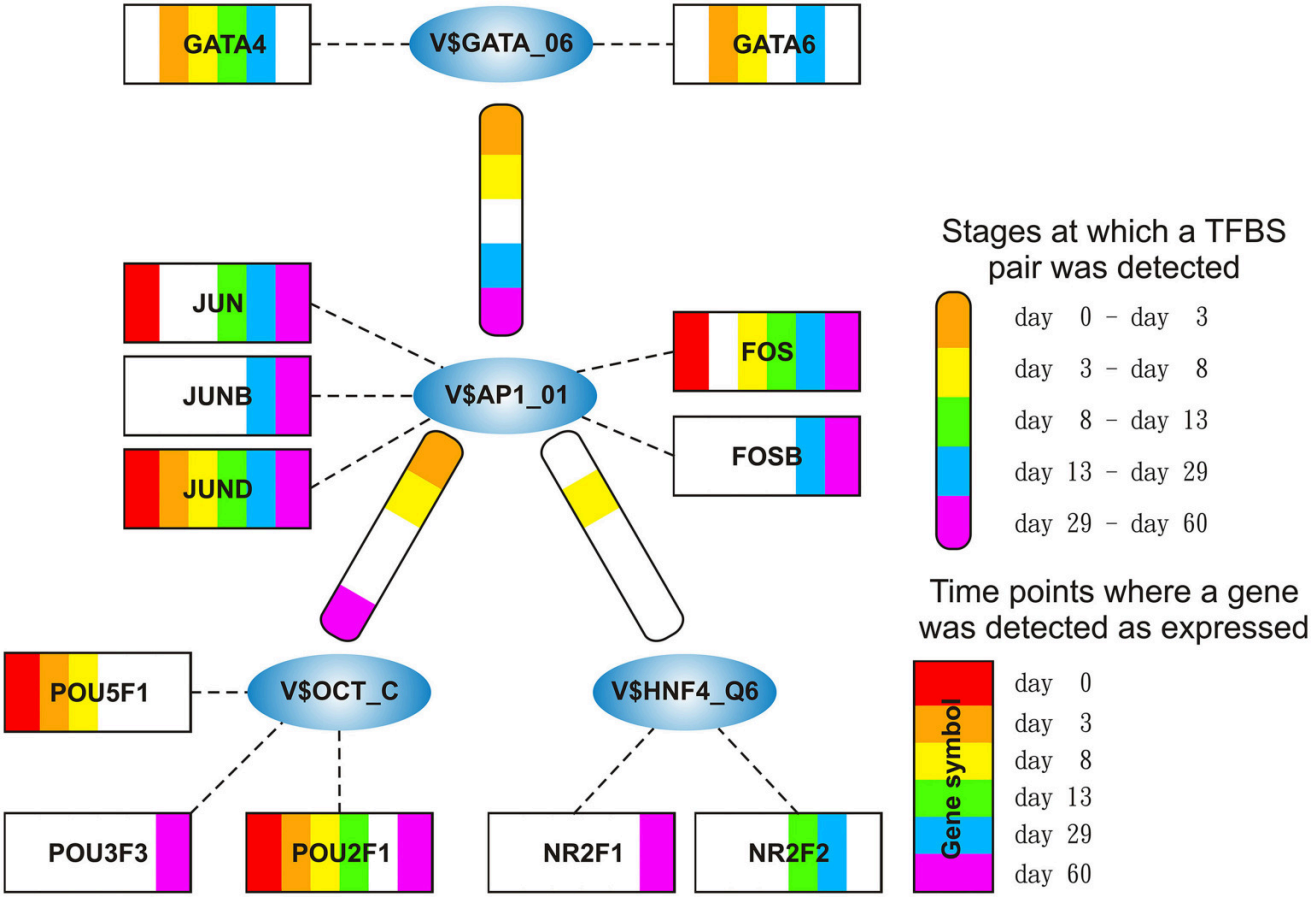
**Stage specific representation of TFBSs and the expression of associated TF genes, referring to Figure 2A**. The encircled nodes represent the found TFBSs which are connected by color-coded round-edged rectangles which highlight stages where a TFBS pair was found. TF genes which are associated to TFBSs are linked by dashed lines. The TF genes are represented by color-coded rectangles representing the presence at a partiular time point. The absence of a TF gene during a particular time point or the absence of a pair during a particular stage is encoded in white. Both, the color-code for the stage specificity as well as for the gene expression of a TF gene is shown on the bottom right side. TF genes which are associated to a TFBS but are in all time points below the set threshold are omitted.

The AP-1-cluster is an assembly of different TFBSs with the V$AP1_01 binding site in its center (see Figure 2A). As described in Table 1 and in Figure 3, V$AP1_01 binding site co-occurs with V$OCT_C binding site during mesoderm induction (< day 3) and early cardiac specification stage (day 3–day 8) and at late cardiac maturation stage (> day 29). Further, V$AP1_01 co-occurs with V$GATA_Q6 binding site at all stages except days 8–13. Interestingly, a co-occurring pair between V$AP1_01 and V$HNF4_Q6 binding site was detected only between day 3 and day 8. Additionally, Figure 3 shows for these TFBSs the related TF genes which are expressed in at least one time point.

**Table 1:** **TFBS pairs within the AP-1-cluster**.

	**Day0–Day3**	**Day3–Day8**	**Day8–Day13**	**Day13–Day29**	**Day29–Day60**
V$AP1_01 − V$OCT_C	+	+	−	−	+
V$AP1_01 − V$GATA_Q6	+	+	−	+	+
V$AP1_01 − V$HNF4_Q6	−	+	−	−	−

*Constitution of co-occurring pairs in the AP-1-cluster, a “+” indicates the presence of a pair; a “−” its absence. During the late stage of cardiac specification (Day8–Day13), the cluster is completely absent*.

**Figure 4:**
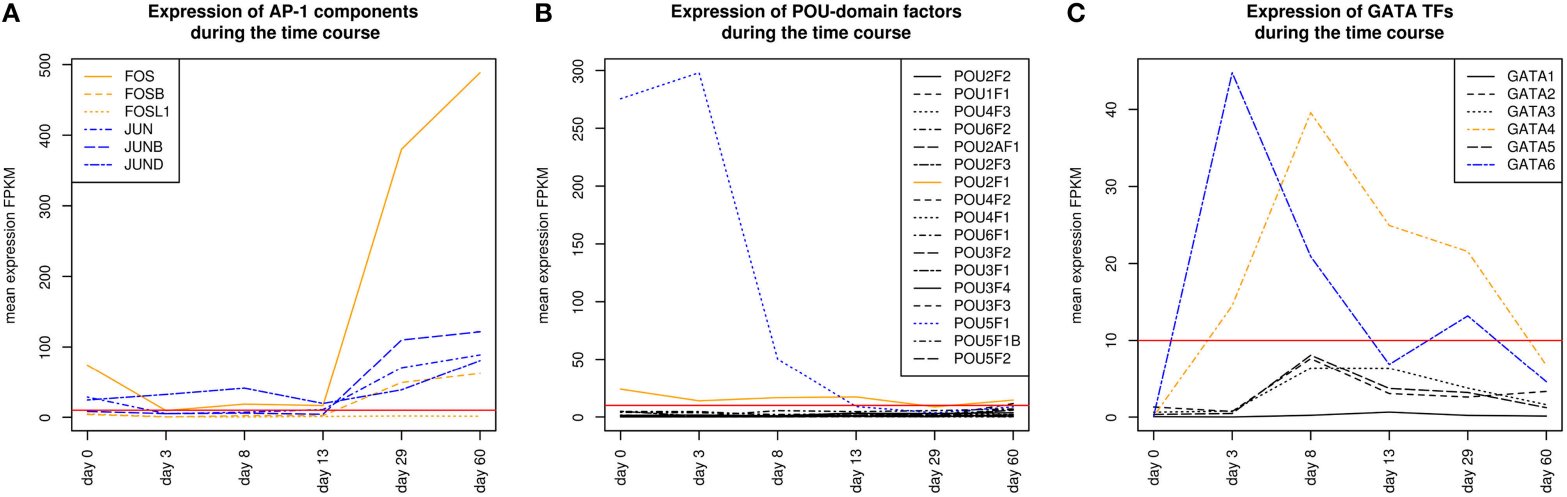
**(A)** Expression of AP-1 factor genes. In orange FOS TF genes are shown, and in blue JUN TF genes. At early stages the expression levels of FOS and JUN genes which are AP-1 components is rather limited. It is likely that AP-1 cannot be formed due to the low expression of FOS genes. In later stages (> day 13), AP-1 and especially FOS increases its expression. **(B)** Expression of TF genes which contain a POU-domain, in blue (POU5F1) and orange (POU2F1) are the two genes which are above the threshold. POU5F1 which is more abundant than POU2F1 decreases during the time course and was absent after day 13. **(C)** Expression of GATA genes, in blue (GATA6) and orange (GATA4) are above the threshold. GATA6 is expressed during the mesoderm induction stage and decreases afterwards, while GATA4 becomes supreme in subsequent stages. The red lines show a FPKM value of 10 that we consider as threshold for sufficiently expressed genes which contribute to regulatory effects.

AP-1 is a family of leucine zipper transcription factors (bZIP) which forms homo- or heterodimers composed of proteins belonging to JUN or FOS protein families (Shaulian and Karin, 2002; Hess et al., 2004; Shaulian, 2010). AP-1 plays a role in the regulation of general functions like proliferation, differentiation, and apoptosis. We identified that V$AP1_01 co-occurs with V$OCT_C binding sites which are bound by AP-1 and POU-domain factors like POU5F1, respectively. POU5F1 is also known as OCT-4, which is an important pluripotency maintenance factor (Schöler et al., 1990; Nichols et al., 1998; Pesce and Schöler, 2001; Guo et al., 2002). Regarding the expression values, POU5F1 shows higher expression in early stages (< day 8) and is absent after day 13 (see Figure 4B). This is in contrast to AP-1, where AP-1 components (FOS as well as JUN) are not present or only present at reduced levels during early stages, but they show increased expression values after day 13 (see Figure 4A). This suggests that AP-1 may not be formed during early stages, where POU5F1 controls the associated genes, and that during the late cardiac maturation stage (> day 29) the analyzed genes are under control of AP-1.

Our analysis identified a co-occurrence of V$AP1_01 with V$GATA_Q6 binding sites. GATA factors form a protein family of six zinc finger transcription factors that share a highly conserved DNA-binding sequence (Orkin, 1992; Ohneda and Yamamoto, 2002; Pikkarainen et al., 2004; Brewer and Pizzey, 2006). As suggested in Brewer and Pizzey (2006), the family can be dissected into two subfamilies (GATA-1,2,3 and GATA-4,5,6), based on their expression levels in different tissues, where only GATA -4, -5 and -6 are associated with cardio- and organogenesis (Pikkarainen et al., 2004; Peterkin et al., 2005; Brewer and Pizzey, 2006; Whitfield et al., 2012; Turbendian et al., 2013). We found only GATA4 and GATA6 to be expressed. Interactions between GATA-factors and AP-1 are well known, especially co-occurrence of AP-1 together with GATA-4 in several heart cell types and in Leydig cells (Herzig et al., 1997; Suzuki et al., 1999; Schröder et al., 2006; Linnemann et al., 2011; Martin et al., 2012). In our system, GATA6 was expressed in high amounts during the mesoderm induction (< day 3) and early cardiac specification stage (day 3–day 8) but was not expressed or only at minor extent during cardiac maturation (> day 13, see Figure 4C). In contrast, GATA4 was expressed in high amounts during the late cardiac specification stage as well as during cardiac maturation (> day 8). The missing of AP-1 during mesoderm induction (< day 3) suggests that genes specific for mesoderm induction might be under control of GATA-6, whereas GATA-4 and AP-1 may regulate genes during cardiac maturation (> day 13), synergistically (see Pikkarainen et al., 2004 for the role of GATA-4 and GATA-6).

The role of the co-occurrence between V$AP1_01 and V$HNF4_Q6, which represents a binding site for HNF4A or HNF4G TFs, during cardiomyogenesis is uncertain. This TFBS pair was detected during early cardiac specification stage (days 3–8), but no expression of the related genes could be found. As mentioned before, the formation of AP-1 during this stage at relevant levels is uncertain (see Figure 4A), due to the low expression of the AP-1 components. Furthermore, the role of HNF4-genes, which where frequently reported to be associated with lipid metabolism in the liver (Watt et al., 2003; Chandra et al., 2013), during cardiac development is still unclear, but may point to changes in the metabolism at this stage.

### 3.2. HMGIY-cluster

**Figure 5:**
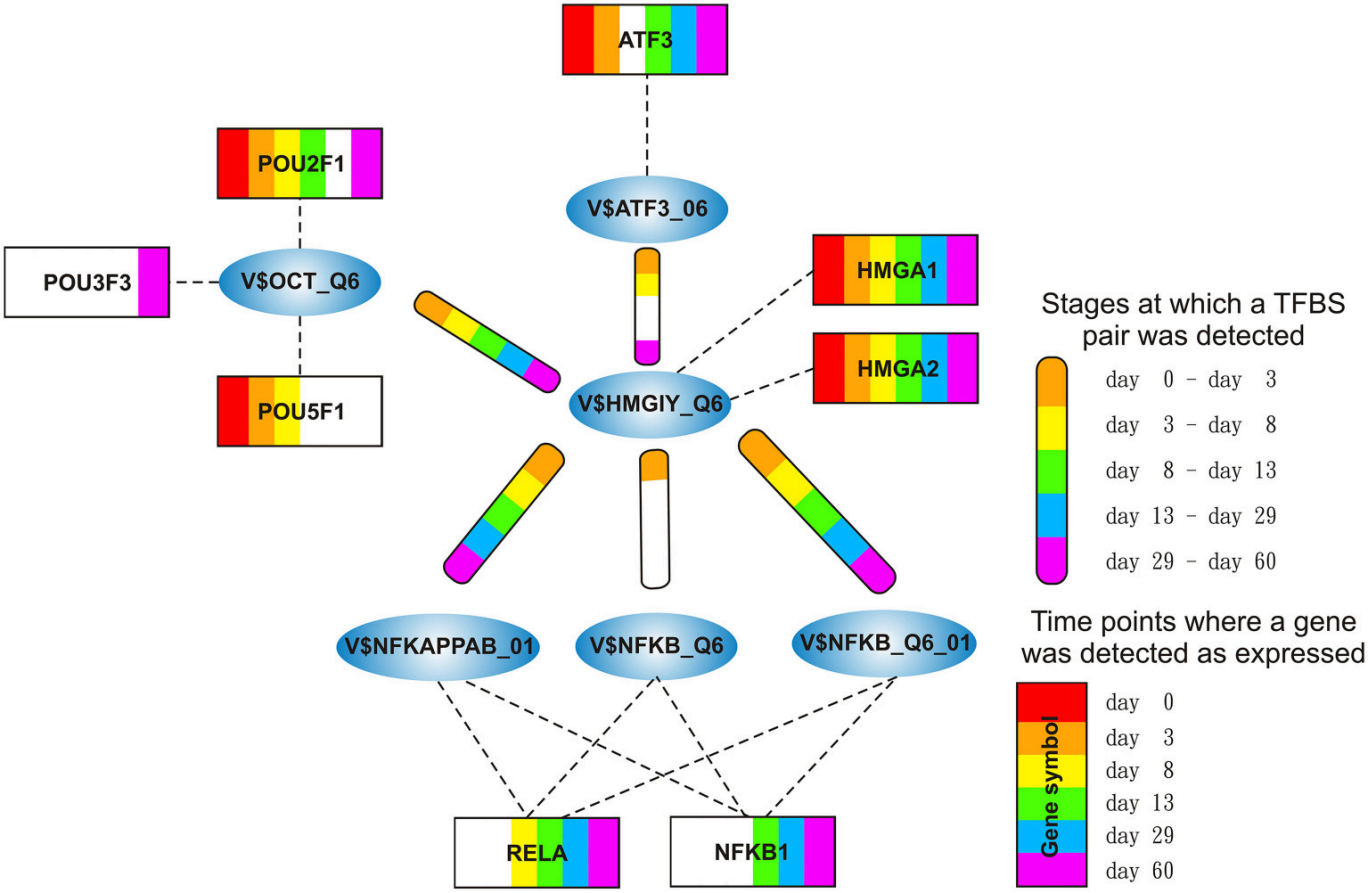
**Stage specific representation of TFBSs and the expression of associated TF genes, referring to Figure 2B**. The encircled nodes represent the found TFBSs which are connected by color-coded round-edged rectangles which highlight stages where a TFBS pair was found. TF genes which are associated to TFBSs are linked by dashed lines. The TF genes are represented by color-coded rectangles representing the presence at a partiular time point. The absence of a TF gene during a particular time point or the absence of a pair during a particular stage is encoded in white. Both, the color-code for the stage specificity as well as for the gene expression of a TF gene is shown on the bottom right side. TF genes which are associated to a TFBS but are in all time points below the set threshold are omitted.

The HMGIY-cluster is assembled in a total of five TFBS pairs (see Figures 2B, 5) with the V$HMGIY_Q6 binding site in its center. Table 2 shows the co-occurring TFBS pairs of this cluster and Figure 5 shows for these TFBSs the related TF genes which are expressed in at least one time point. The TFBS pair V$HMGIY_Q6 - V$OCT_Q6 was found during all stages and the co-occurrence between V$HMGIY_Q6 and V$ATF3_Q6 binding sites was found at days 3–8, and after day 29. Interestingly, we found in this cluster three binding sites, namely V$NFKAPPAB_01, V$NFKB_Q6_01, and V$NFKB_Q6 which can be bound by the family of NF-κB-related factors. While the V$HMGIY_Q6 - V$NFKB_Q6 TFBS pair was detected only during the mesoderm induction stage (< day 3), the co-occurrence between V$HMGIY_Q6 and V$NFKB_Q6_01 binding sites was found at all stages. The TFBS pair V$HMGIY_Q6 - V$NFKAPPAB_01 was found at all stages except the late cardiac specification stage (day 8–day 13). To ensure the quality of these three NF-κB binding sites, we further investigated their position weight matrices (PWMs) as well as their binding motifs. Considering the PWMs, we observed that all PWMs have relatively high value of information content (see Table 3) which assess their quality. In addition, a comparison between motifs shows different binding behavior of NF-κB-related factors which could be linked to specific members of this family.

**Table 2:** **TFBS pairs within the HMGIY-cluster**.

	**Day0–Day3**	**Day3–Day8**	**Day8–Day13**	**Day13–Day29**	**Day29–Day60**
V$HMGIY_Q6 − V$OCT_Q6	+	+	+	+	+
V$HMGIY_Q6 − V$NFKAPPAB_01	+	+	−	+	+
V$HMGIY_Q6 − V$NFKB_Q6_01	+	+	+	+	+
V$HMGIY_Q6 − V$NFKB_Q6	+	−	−	−	−
V$HMGIY_Q6 − V$ATF3_Q6	+	+	−	−	+

*Constitution of co-occurring pairs within the HMGIY-cluster, a “+” indicates the presence of a matrix pair; a “−” its absence*.

**Table 3:** **Binding sites for different NF-κB PWMs found in the HMGIY-cluster**.

**PWM**	**Information content**	**Motif**
V$NFKAPPAB_01	11.8	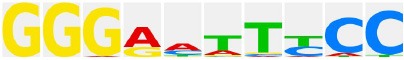
V$NFKB_Q6_01^(*rc*)^	13.3	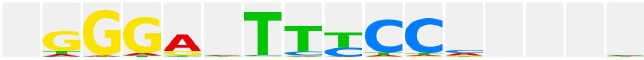
V$NFKB_Q6	14.4	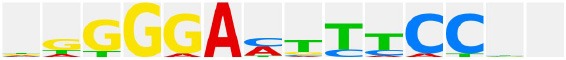

The family of NF-κB-related factors can be represented by different PWMs each of which have relatively high information content and different binding motifs. ^(rc)^: reverse complement

**Figure 6:**
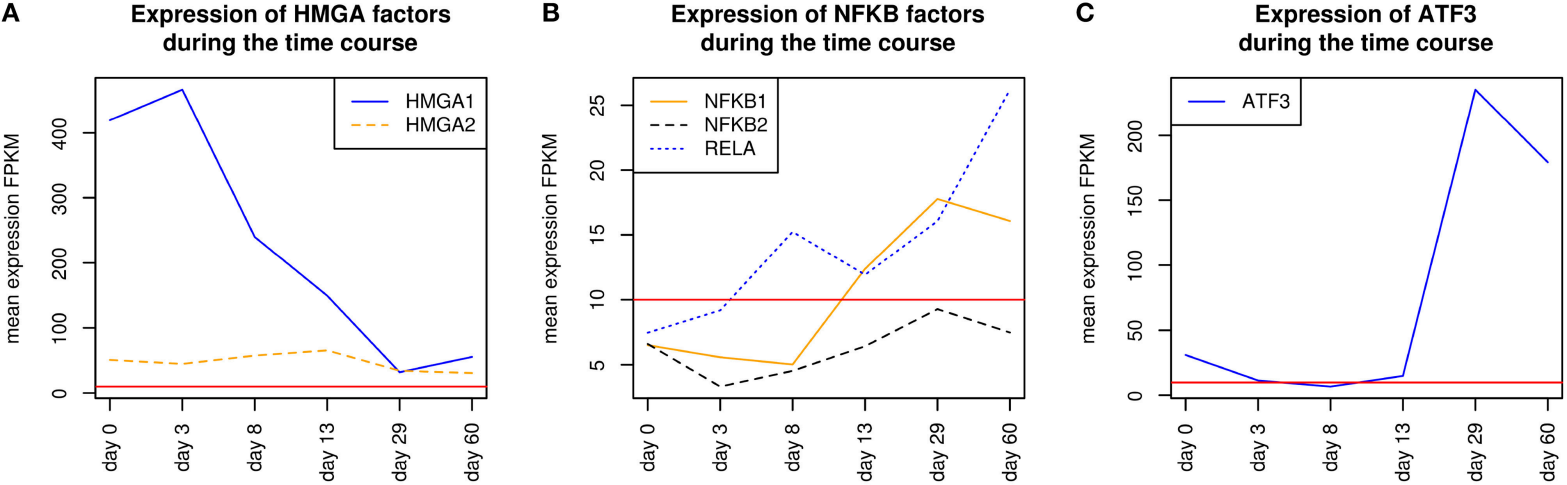
**Expression of corresponding TF genes which can be represented by the PWM V$OCT_Q6 have been shown in Figure 4B**. **(A)** Expression of corresponding TF genes which can be represented by the PWM V$HMGIY_Q6. HMGA1 is dominant over HMGA2 but decreases during the time course. **(B)** Expression of corresponding TF genes which can be represented by the PWMs (V$NFKAPPAB_01, V$NFKB_Q6_01, V$NFKB_Q6) related to NF-κB binding sites. While NFKB2 never reaches the threshold, RELA and NFKB1 increase their expression levels in later stages (> day 13). **(C)** Expression of the corresponding ATF3 gene to its TF which can be represented by the PWM V$ATF3_Q6. ATF3 is present in the first stage, but in subsequent stages until day 13 it is quite close to the threshold. It changes its expression levels drastically during the cardiac maturation stage (> day 13). The red lines show a FPKM value of 10 that we consider as threshold for sufficiently expressed genes which contribute to regulatory effects.

HMGA1 is a TF which is represented by the PWM V$HMGIY_Q6 and was recently described as a positive regulator of pluripotency in cellular reprogramming (Shah et al., 2012). The expression levels of HMGA1 in our system are in agreement with previous studies, which describe HMGA1 as highly abundant during embryogenesis, especially in embryonic stem cells; with intermediate expression levels in undifferentiated cancers and at low or at not detectable levels in adult differentiated cells and fibroblasts (Fusco and Fedele, 2007; Hillion et al., 2008, 2009; Resar, 2010; Chou et al., 2011; Schuldenfrei et al., 2011; Shah et al., 2012; Williams et al., 2015). The detected co-occurrence between V$HMGIY_Q6 and V$OCT_Q6 binding sites was found at all stages. The corresponding TF genes (HMGA1, HMGA2, and POU5F1) of this TFBS pair did not show such behavior (see Figures 4B, 6A). HMGA1 as well as POU5F1 are expressed at high levels during early cardiac development with their maximum expression levels at day 3 and declined afterwards. However, this pair was found at later stages indicating that the detected DEGs at these stages could be potentially regulated by this pair. POU5F1 is below the threshold after day 13, whereas HMGA1 is always above the threshold but stablized at low levels. After day 13, HMGA1, which is in its expression values always more abundant than HMGA2, could regulate the detected pairs alone.

The co-occurrence of V$HMGIY_Q6 and different NF-κB binding sites was detected at all time points (see Table 2). Interestingly, our findings show that this interaction could occur based on different NF-κB binding sites which are bound by the same TFs. It is known that the interaction between HMGA1 and NF-κB plays a pivotal role in formation of an enhancer complex which is essential to regulate interferon-β signaling on genomic level (Thanos and Maniatis, 1992; Lewis et al., 1994; Wood et al., 1995; Himes et al., 1996; Thanos and Maniatis, 1996; Mantovani et al., 1998; Perrella et al., 1999; Zhang and Verdine, 1999). Within this complex, NF-κB acts on the one hand as a key regulator in hypertrophy and, on the other hand it acts as cardioprotective factor during embryogenesis (Dewey et al., 2011; Gordon et al., 2011; Liu et al., 2012; Zhou et al., 2013). The expression levels of NF-κB genes may indicate an increasing importance of NFKB1 and especially of RELA during cardiac maturation (> day 13), where it is expressed at considerable levels (see Figure 6B).

The co-occurrence of V$HMGIY_Q6 with the V$ATF3_Q6 binding site, which is bound by ATF3, was detected during early cardiac development until day 8 and at the latest stage after day 29. ATF-3 is a FOS-related TF, which contains a basic leucine zipper as structural motif (Chen et al., 1994). ATF-3 acts as homo- or heterodimer to activate or to repress the expression of target genes, depending on its environment. Further, it is also involved in TGF-β signaling in several cell types and in cardiac development (Ishiguro et al., 2000; Mayr and Montminy, 2001; Yan et al., 2005; Gilchrist et al., 2006; Yin et al., 2010; Lin et al., 2014). While HMGA1 is expressed at high levels during early stages (days 0–3) and is declined afterwards, the ATF3 gene is close to the threshold before day 13 and increases its expression levels during subsequent stages (see Figure 6C). Our results suggest that the genes regulated by this pair are under control of HMGA1 in the early stages and ATF-3 afterwards. Gilchrist et al. demonstrate the co-occurrence of ATF-3 and NF-κB binding sites in regulated target genes (Gilchrist et al., 2006). According to their binding sites, our analysis suggests that together with ATF-3 and NF-κB factor, HMGA1 may play an important role in the regulation of target genes in cardiac development.

### 3.3. SMAD-cluster

**Figure 7:**
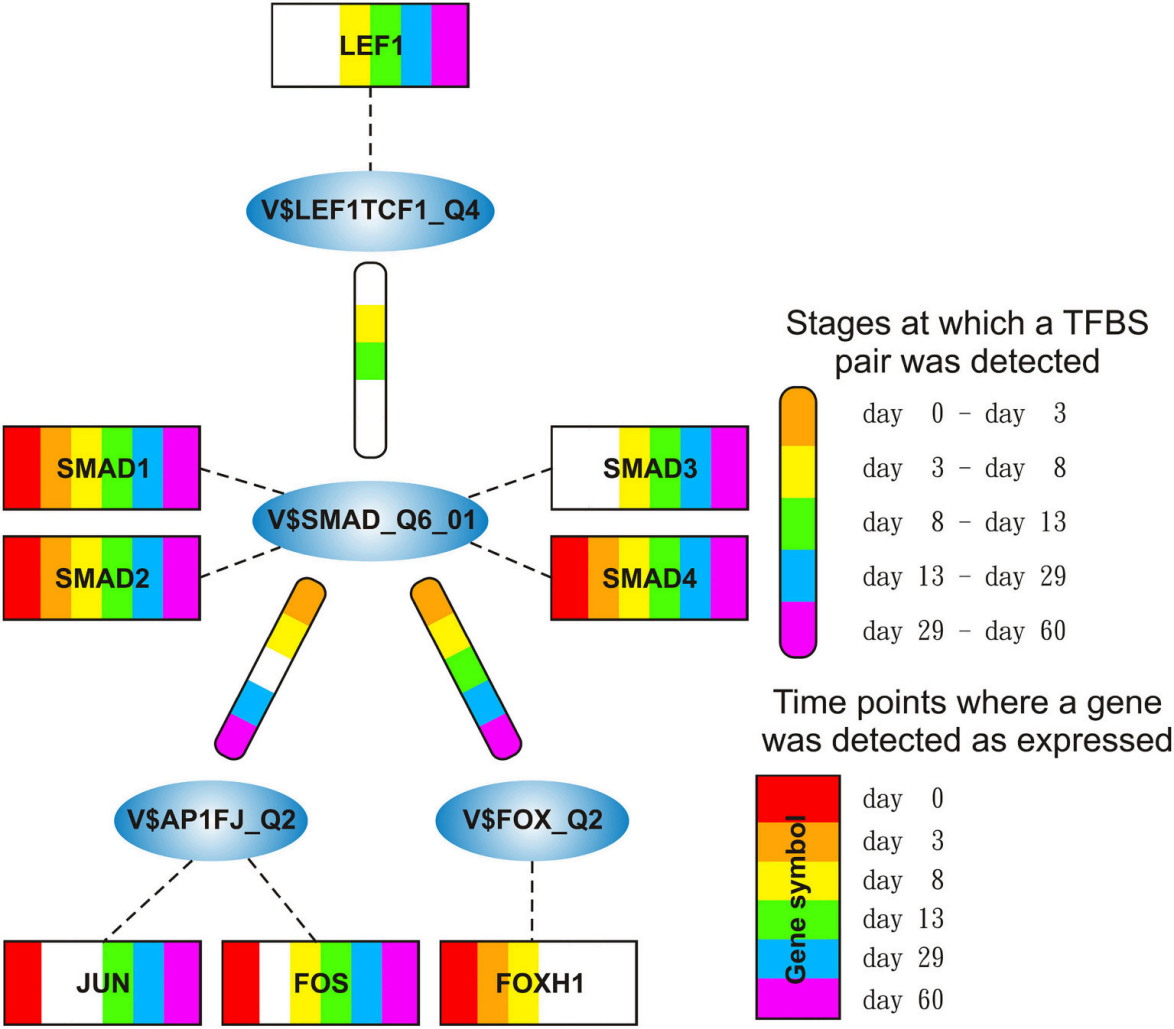
**Stage specific representation of TFBSs and the expression of associated TF genes, referring to Figure 2C**. The encircled nodes represent the found TFBSs which are connected by color-coded round-edged rectangles which highlight stages where a TFBS pair was found. TF genes which are associated to TFBSs are linked by dashed lines. The TF genes are represented by color-coded rectangles representing the presence at a partiular time point. The absence of a TF gene during a particular time point or the absence of a pair during a particular stage is encoded in white. Both, the color-code for the stage specificity as well as for the gene expression of a TF gene is shown on the bottom right side. TF genes which are associated to a TFBS but are in all time points below the set threshold are omitted.

The SMAD-cluster is assembled in a total of three TFBS pairs with the V$SMAD_Q6_01 binding site in its center (see Figures 2C, 7). Table 4 shows the co-occurrence of V$SMAD_Q6_01 and V$FOX_Q2 binding sites in the promoters of the regulated genes and was observed during all stages. The TFBS pair V$SMAD_Q6_01 - V$AP1FJ_Q2 was detected in our system at early stages until day 8 and at late stages after day 13, but not during late cardiac specification stage (days 8–13). In contrast, the co-occurrence between V$SMAD_Q6_01 and V$LEF1TCF1_Q4 was detected only during cardiac specification (days 3–13). In addition, Figure 7 shows for these TFBSs the related TF genes which are expressed in at least one time point.

**Figure 8:**
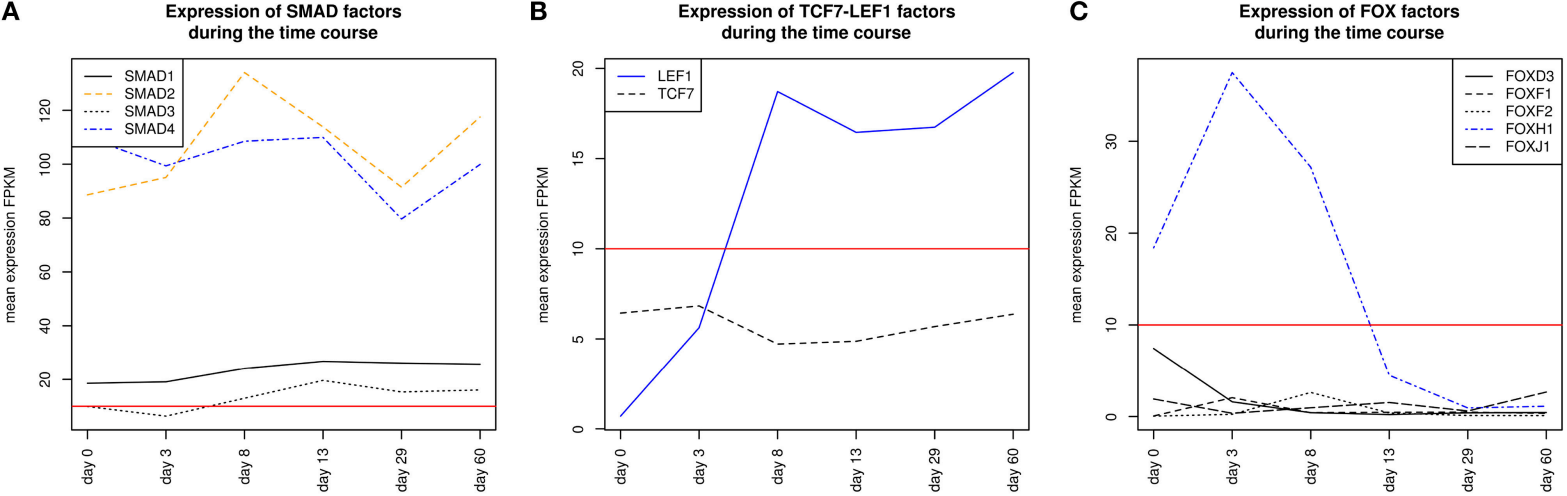
**Expression of corresponding TF genes which can be represented by the PWM V$AP1FJ_Q2 have been shown in Figure 4A.** **(A)** Expression of corresponding genes to TFs which can be represented by the PWM V$SMAD_Q6_01. Each SMAD is expressed during the complete time course at similiar levels, while the expression levels of SMAD2/4 are higher than the expression levels of SMAD1/3. After beginning of the cardiac specification (> day 3) SMAD4 is slightly more abundant than SMAD2 and remains in this position. **(B)** Expression of corresponding TF genes which can be represented by the PWM V$LEF1TCF1_Q4, TCF7 is below the threshold set by us as a limit for robust transcription while LEF1 is clearly transcribed after the mesoderm induction stage (> day 3). The SMAD-TCFLEF-pair was found during the cardiac specification stage only (day 3–day 8). **(C)** Expression of corresponding TF genes which can be represented by the PWM V$FOX_Q2. FOXH1 is the only expressed gene and present until day 13. The red lines show a FPKM value of 10 that we consider as threshold for sufficiently expressed genes which contribute to regulatory effects.

SMADs are members of a family of transcription factors that form a beta-hairpin structure which interacts with the major groove of the DNA (Burke et al., 1976; Macias et al., 2015). SMAD1-4 which can be represented by the PWM V$SMAD_Q6_01 act as TFs in the nucleus and as signaling molecules, where they are involved in numerous pathways like canonical and non-canonical SMAD-signaling pathways, TGF-β- as well as BMP- and WNT-signaling (Heldin et al., 1997; Leask and Abraham, 2004; Euler-Taimor and Heger, 2006; Pal and Khanna, 2006; Schröder et al., 2006; Leask, 2007; Ruiz-Ortega et al., 2007; Calvieri et al., 2012; Massagué, 2012; Dyer et al., 2014; Euler, 2015). Figure 8A shows that SMAD1, SMAD2, and SMAD4 genes are continuously expressed at all stages. The detected SMAD3 expression after day 3 exceeds the set threshold only slightly. SMAD2 and SMAD4 show the highest expression levels in our system, but the differences in their expression levels are rather small.

**Table 4:** **TFBS pairs within the SMAD-cluster**.

	**Day0–Day3**	**Day3–Day8**	**Day8–Day13**	**Day13–Day29**	**Day29–Day60**
V$SMAD_Q6_01− V$FOX_Q2	+	+	+	+	+
V$SMAD_Q6_01 − V$AP1FJ_Q2	+	+	−	+	+
V$SMAD_Q6_01 − V$LEF1TCF1_Q4	−	+	+	−	−

*Constitution of co-occurring pairs within the SMAD-cluster, a “+” indicates the presence of a pair; a “−” its absence*.

The co-occurrence of V$SMAD_Q6_01 and V$FOX_Q2 binding sites was detected at all stages (see Table 4). Recently, the cooperative regulatory interaction of FOX factors, which play an important role in cardiovascular development and in other organs (Yamagishi et al., 2003; Maeda et al., 2006; Seo and Kume, 2006; Fortin et al., 2015), with SMAD3 and SMAD4 has been shown by (Fortin et al., 2015). Although the SMAD-FOX pair can be detected during the whole time course, the expression of FOX-genes is limited to FOXH1, which seems to play a role in early heart development only (< day 13, see Figure 8C).

The co-occurrence between V$SMAD_Q6_01 and V$AP1FJ_Q2 binding sites were found in almost all stages except for the late cardiac specification stage (between day 8 and day 13). In adult CMs, AP-1 together with SMAD proteins modulates hypertrophic, apoptotic and fibrotic pathways. Additionally, AP-1 together with SMAD forces the shift toward apoptosis after stimulation of TGF-β-signaling (Schneiders et al., 2005; Schröder et al., 2006; Euler, 2015). In the embryonic hearts, the activation of TGF-β-pathways results in an induction of cardioprotective functions (Leask and Abraham, 2004; Pal and Khanna, 2006; Leask, 2007; Ruiz-Ortega et al., 2007; Calvieri et al., 2012; Euler, 2015). Although there is no known AP-1 SMAD interaction during cardiogenesis, Yuan et al., shows the interaction of these TFs by usage of AP-1 and SMAD decoy oligodeoxynucleotides, which reduces fibrosis in their study (Yuan et al., 2013).

The detected TFBS pair V$SMAD_Q6_01 - V$LEF1TCF1_Q4 is limited to the cardiac specification stage (day 3–day 13). TCF-7 and LEF-1 transcription factors, which are represented by V$LEF1TCF1_Q4, can be activated by β-catenin and are involved in canonical WNT-signaling (Brade et al., 2006; Chen et al., 2006; Pal and Khanna, 2006; Kwon et al., 2007; Naito et al., 2010). The measured gene expression of TCF as well as LEF genes shows that during cardiac specification both groups are quite close to or below the set threshold (see Figure 8B). This indicates that no TCF or LEF binding occurs, which may result in the absence of canonical WNT-signaling during cardiac specification.

### 3.4. NFAT-cluster

**Figure 9:**
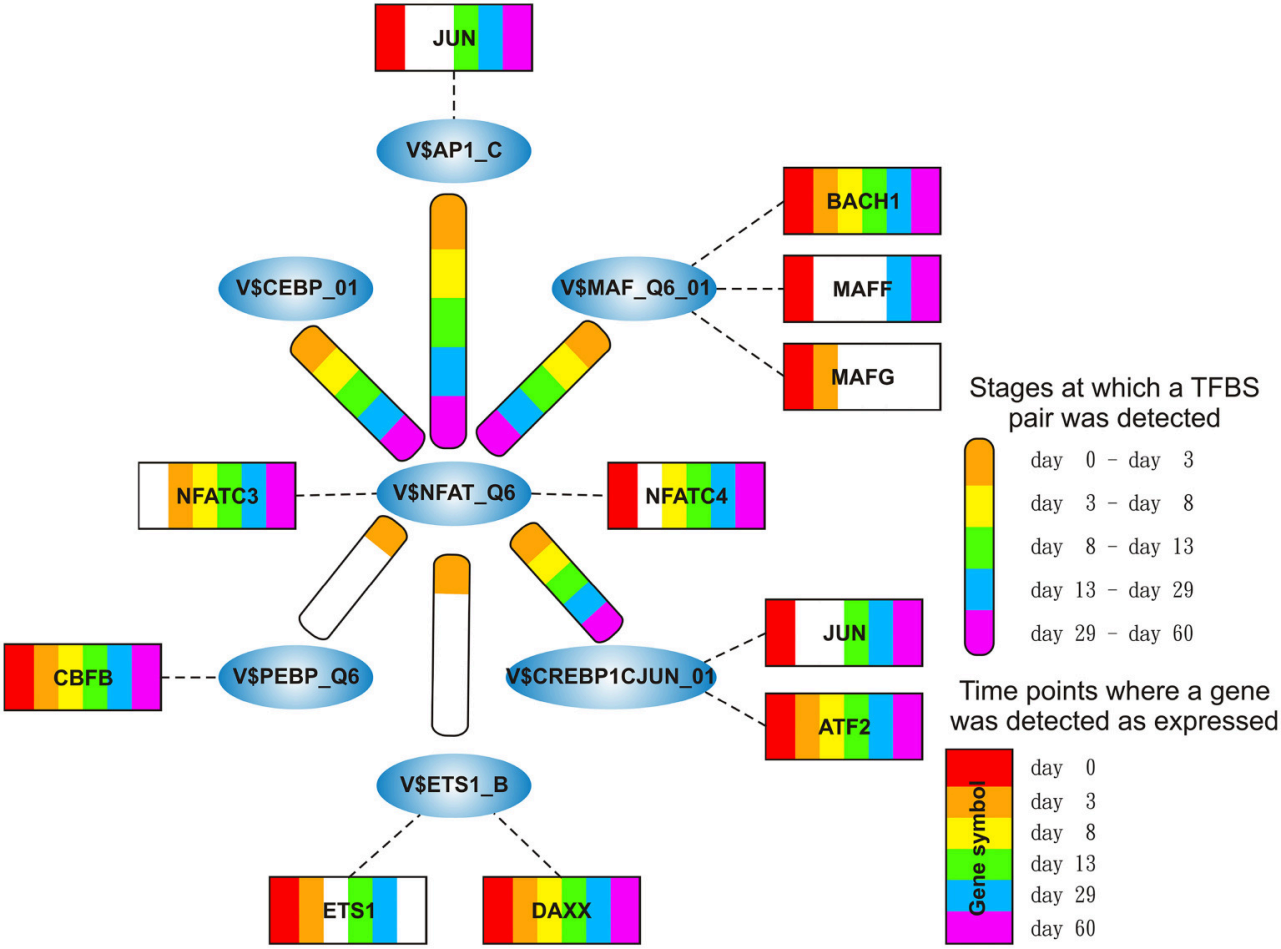
**Stage specific representation of TFBSs and the expression of associated TF genes, referring to Figure 2D**. The encircled nodes represent the found TFBSs which are connected by color-coded round-edged rectangles which highlight stages where a TFBS pair was found. TF genes which are associated to TFBSs are linked by dashed lines. The TF genes are represented by color-coded rectangles representing the presence at a partiular time point. The absence of a TF gene during a particular time point or the absence of a pair during a particular stage is encoded in white. Both, the color-code for the stage specificity as well as for the gene expression of a TF gene is shown on the bottom right side. TF genes which are associated to a TFBS but are in all time points below the set threshold are omitted.

The NFAT-cluster consists in a total of six TFBS pairs with V$NFAT_Q6 binding site in its center (see Figures 2D, 9). As described in Table 5 and Figure 9, V$NFAT_Q6 co-occurs with V$PEBP6_Q6 and V$ETS1_B binding sites only during the mesoderm induction stage (days 0–3). Three TFBS pairs, namely V$NFAT_Q6 - V$AP1_C, V$NFAT_Q6 - V$CREBP1CJUN_01, and V$NFAT_Q6 - V$MAF_Q6_01, were found during the complete time course. The co-occurrence of V$NFAT_Q6 with V$CEBPB_01 binding sites in the promoter regions of the analyzed set of genes was found as present until day 8 and during the cardiac maturation stage after day 13. This TFBS pair was not present during the late cardiac specification stage (days 8–13). In addition, Figure 9 shows for these TFBSs the related TF genes which are expressed in at least one time point.

**Table 5:** **TFBS pairs within the NFAT-cluster**.

	**Day0–Day3**	**Day3–Day8**	**Day8–Day13**	**Day13–Day29**	**Day29–Day60**
V$NFAT_Q6 − V$PEBP_Q6	+	−	−	−	−
V$NFAT_Q6 − V$AP1_C	+	+	+	+	+
V$NFAT_Q6 − V$CEBPB_01	+	+	−	+	+
V$NFAT_Q6 − V$CREBP1CJUN_01	+	+	+	+	+
V$NFAT_Q6 − V$MAF_Q6_01	+	+	+	+	+
V$NFAT_Q6 − V$ETS1_B	+	−	−	−	−

*Constitution of the NFAT-cluster, a “+” indicates the presence of a matrix pair; a “−” its absence*.

**Figure 10:**
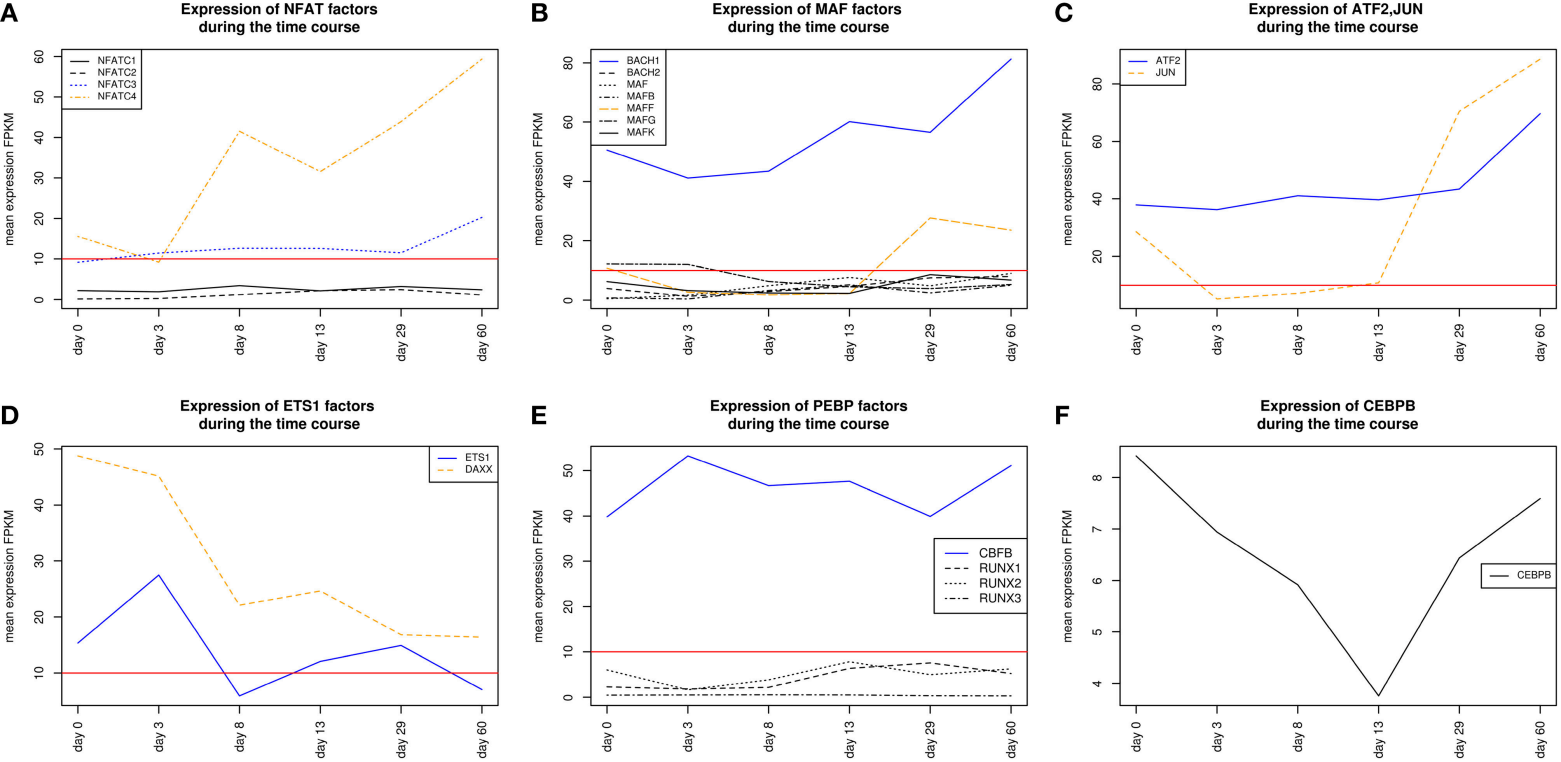
**Expression of TF genes corresponding to their factors and represented by the PWM V$AP1_C have been shown in Figure 4A**. **(A)** Expression of TF genes which corresponds to NFAT factors represented by PWM V$NFAT_Q6. NFATC3 and NFATC4 are above the set threshold, whereas NFATC3 is more abundant than NFATC4. **(B)** Expression of TF genes which can be represented by the PWM V$MAF_Q6_01. MAFB shows expression levels slightly above the threshold set by us as a limit for robust transcription during the mesoderm induction stage while MAFF is expressed during cardiac maturation (> day 13). BACH1 is found to be expressed during the complete time course at considerable levels and is always more abundant than all other TF genes, which corresponds to V$MAF_Q6_01. Additionally, BACH1 increases its expression value after the mesoderm induction stage (> day 3). **(C)** Expression of TF genes which can be represented by the PWM V$CREBP1CJUN_01. ATF2 is expressed during the complete time course and increases its expression value in the latest stage. JUN is expressed at day 0 and after day 13 where it exceeds the expression levels of ATF2. **(D)** Expression of TF genes which can be represented by the PWM V$ETS1_B. DAXX is expressed during all time points, but its expression diminishes continuously. Nevertheless, it shows expression levels which are always above ETS1. **(E)** Expression of TF genes which can be represented by the PWM V$PEBP_Q6. Only CBFB shows expression above the threshold and was found as continuously expressed. **(F)** Expression of CEBPB which can be represented by the PWM V$CEBPB_01. CEBPB is during the complete time course below the set threshold and is considered to be low or not expressed. The red lines show a FPKM value of 10 that we consider as threshold for sufficiently expressed genes which contribute to regulatory effects.

Regulatory roles for NFAT factors, which can be represented by the PWM V$NFAT_Q6, have been discovered in diverse organs and cells, including the central nervous system, blood vessels, heart, skeletal muscle and haematopoietic stem cells (Macián, 2005). In general, an activation of factors of the NFAT family is calcium dependent and has been described to be of specific importance in development of the atrial myocardium and the morphogenesis of heart valves (Graef et al., 2001; Crabtree and Olson, 2002; Schubert et al., 2003; Schulz and Yutzey, 2004). In our system, only NFATC3 and NFATC4 showed expression levels above the threshold. Comparing the expression levels, NFATC4 is more abundant than NFATC3 at all time points, except for day 3, but both genes increase their expression levels at later stages and especially after day 29 (see Figure 10A).

The detected co-occurrence of TFBS pairs V$NFAT_Q6 - V$AP1_C and V$NFAT_Q6 - V$PEBP_Q6 refers either to NFAT-AP-1 or to NFAT-RUNX interactions which have been mainly observed in the immune system (Macián, 2005). Macián et al. have demonstrated that the interaction between NFAT and AP-1 can be linked to calcineurin dependent pathways as well as to regulation of MAP kinase pathways (Macián et al., 2001). Additionally, NFAT and AP-1 cooperate in naïve T-cells with RUNX TFs as well as with NF-κB in the promoter of IL-2 during T-cell activation (see Figures 10C,E) (Hermann-Kleiter and Baier, 2010). In our system, the low or absent expression of RUNX indicates no relevance for these factors. However, the corresponding binding site can be also occupied by CBFB, which is associated to congenital heart anomalies and is expressed during all time points (Khan et al., 2006).

We found the co-occurring TFBS pair V$NFAT_Q6 - V$MAF_Q6_01 at all stages. For the corresponding factors it has been shown by Hogan et al. that NFAT factors and MAF were able to activate IL-4 promoters (Hogan et al., 2003). Of all TFs linked to V$MAF_Q6_01, BACH1 is expressed at all stages and is always more abundant than the other genes shown in Figure 10B. This suggests a synergistic interaction in gene regulation between these factors during the complete time course. Furthermore, the interaction between NFAT and MAF factors was observed simultaneously at classical NFAT-AP-1 interaction sites (Hogan et al., 2003).

The co-occurrence between V$NFAT_Q6 and V$CEBPB_01 binding sites has been described in liver cell lines by Yang and Chow (2003). The corresponding factors to this pair seem to interact in a formation of a composite enhancer complex (Yang and Chow, 2003). In our system, genes that are linked to V$CEBPB_01 binding sites are not expressed (see Figure 10F). The observation of this pair and its potential role in heart development remains unclear.

The role of the TFBS pair V$NFAT_Q6 - V$ETS1_B, which was detected during the mesoderm induction stage, remains unclear. ETS1, a TF gene which can be linked to the PWM V$ETS1_B, is required for the differentiation of cardiac neural crest (Gao et al., 2010). Although ETS1 was expressed during the mesoderm induction stage (days 0–3), its expression is markedly reduced afterwards. DAXX is another gene that is linked to the PWM V$ETS1_B and is at all time points more abundant than ETS1 (see Figure 10D). The DAXX factor inhibits apoptosis in cardiac myocytes (Zobalova et al., 2008). An interaction between NFAT and DAXX was not found in literature, and thus the role of this pair remains unclear.

## 4. Discussion

Today, it is known that in higher organisms transcription factors have to interact with each other to regulate gene expression which leads to a proper development of tissues and organs. So far, several studies have shown that the co-occurence of TF binding sites (TFBSs) on sequences is an essential indication for the identification of interactions between TFs. In this study, we identified co-occurring TFBS pairs by applying MatrixCatch algorithm to the promoter regions of five differentially expressed gene sets, which are based on a time course dataset of developing human myocardium, modeled in a tissue engineering approach (Hudson et al., in revision). MatrixCatch is a statistically affirmed computational method for the recognition of experimentally verified interactions between TFs according to their TFBS localizations in promoters. However, MatrixCatch recognizes based on its underlying algorithm all detectable TFBS pairs of known interacting TFs in promoter regions. This results in a huge overlap between recognized pairs at different stages, although these pairs can play different roles for each stage. To eliminate this drawback of MatrixCatch to some extent, we created an interaction network based on the TFBS pairs for each stage and then applied the MCL algorithm. MCL differentiates negligible TFBS pairs from densely connected TFBS pairs within these interaction networks and thus determines clusters of TFBSs. Such clusters are important to highlight stage specific co-occurrences of TFBS pairs which provide essential knowledge in the understanding of molecular mechanism of cardiac development.

Additionally, we applied our approach to different lengths of putative promoter regions ([from −500 bp to 0], [from −500 bp to +100 bp], [from −1000 bp to 0]) to determine the influence of promoter lengths on the composition of stage-specific clusters. The results denote that there is a considerably high overlap between stage-specific clusters derived from different putative promoter regions (data not shown). Thus, we considered the -1 kb putative regulatory promoter region for our analysis, which is consistent with our experience and provides the most reliable results.

Although, we filtered MatrixCatch outputs using MCL algorithm to reduce weak co-occurrence of TFBSs in each stage, we detected in our analysis several clusters as well as TFBS pairs whose potential role during cardiac development are unclear. One possible reason for the detection of such pairs could depend on the underlying methodology of MatrixCatch. It uses a computational prediction approach which scans promoter sequences and their reverse complements to identify TFBSs using PWMs. However, computational identifications of TFBSs generally suffer from high rates of false positive predictions. Another reason for the detection of those clusters or pairs could be due to genes which are expressed at high levels but play different roles in different tissues. As a result, we could identify such clusters or pairs that might play important roles in the regulation of those genes in other tissues but not in heart. For example, we identified the TFBS pair (V$NFAT_Q6 - V$CEBPB_01) in the NFAT-cluster whose importance has been shown by Yang and Chow in liver (Yang and Chow, 2003), but the potential role of this pair during the cardiac development is unclear. In this context, we also observed the ETS cluster with the V$ETS_Q6 binding site in its center (see Supplementary File 4). Only some individual components, like ETS factors, in this cluster are associated with potential cardiac functionalities. However, considering TFBS pairs in the ETS cluster, we cannot verify their potential role during the cardiac development.

Our results suggest that different types of co-occurring TFBS pairs can be assigned into two main categories: (i) TFBS pairs which are present in the beginning and in later stages but absent in at least one of the subsequent stages; (ii) TFBS pairs which are present during all stages. In our clusters presented in the Result section, there are different co-occurring TFBS pairs, like V$AP1_01 - V$OCT_C and V$HMGIY_Q6 - V$ATF3_Q6, which fall into the first category. Considering the expression values of TF genes for those pairs, we observed that one TF gene was highly expressed in the beginning stages while its partner is expressed at low levels. After the re-occurrence of such a pair in later stages, the measured expression values of TF genes are exactly the opposite. Consequently, the related TFs cannot act in a synergistic manner but rather in an antagonistic manner. Very drastically, we observed this situation in the expression of AP-1 components and POU5F1, which can be linked to V$AP1_01 - V$OCT_C TFBS pair (see Figures 4A,B). Due to this finding we hypothesize that further TFBS pairs, which fall into the first category, could be helpful to enhance our knowledge on the combinatorial code underlying transcriptional regulation of cardiomyogenesis.

This findings could be discussed in the perspective of the “embryonic hourglass“ which describes high divergence in the embryonic shape of vertebrates, insects, like *Drosophila*, and plants, in early and late developmental stages, but minor divergence in mid-stages (Duboule, 1994; Raff and Wolpert, 1996; Kalinka et al., 2010; Quint et al., 2012). In our study, the number of DEGs as well as the number of identified clusters is high in early stages, converge to a minimum during the late cardiac specification stage (day 8–day 13) and increase afterwards again, which is consistent with the general structure of the hourglass model. Furthermore, the identified TFBS pairs, which fall into the first category, could be separated into two different subsets of genes, the one subset is up-regulated before the late cardiac specification stage, while the other subset is up-regulated afterwards and is supposed to regulated cardiac maturation processes. Our findings support the hourglass model derived by previous findings in *Arabidopsis* as well as several animals (Domazet-Lošo and Tautz, 2010; Kalinka et al., 2010; Quint et al., 2012).

In contrast to the TFBSs pairs in the first category, the co-occurrence of TFBS pairs that fall into the second category seems to indicate a synergistic cooperation between related TFs. In our presented clusters, we obtained several TFBS pairs like V$HMGIY_Q6 - V$OCT_Q6, V$SMAD_Q6_01 - V$FOX_Q2, and V$NFAT_Q6 - V$CREBP1CJUN_01 (for detail see Tables 2–4). Considering the expression values of corresponding TF genes for those pairs, we determined that these genes are regulated similarly. For instance, the TF genes HMGA1 and POU5F1, which are linked to V$HMGIY_Q6 and V$OCT_Q6, respectively, are highly expressed during first developmental stages and diminish their levels after day 3. This condition is also observed for the TFBS pair V$NFAT_Q6 - V$CREBP1CJUN_01 where the associated TF genes are expressed at low levels in the beginning and increase their expression levels in later stages.

Altogether, in our study we performed a systematic analysis of TFBS pairs to address the question of cooperation between TFs linked to TFBS pairs, which could play a crucial role through five different cardiac developmental stages. Addressing this question, our results show that some TFBS pairs can be detected at all developmental stages. Furthermore, we obtained the same TFBS pairs at very early and very late stages of the differentiation, although these stages are completely different in their functions. Especially considering expression values of related TF genes of these pairs, we determined that co-occurrence between TFBSs does not always indicate a synergistic regulation of target genes. This finding suggests that corresponding TFs of these pairs can be bound in a mutual exclusive manner, which is important during cardiac development to differentiate between stem cell programs and later embryogenic programs.

## 5. Conclusion

We identify transcription factor pairs that drive cardiac development from stem cells to mature cells in a 60 day time course dataset. Our approach is motivated by the importance of potentially interacting transcription factors represented by the co-occurrence of their TFBSs in the regulated stages specific genes and their mediated effects. We identified the relevant pairs employing MatrixCatch method with Markov clustering algorithm together to highlight stage specific clusters of co-occurring TFBS pairs. Furthermore, we analyzed the changes within these clusters to show the specificity of the gene regulation in cardiac development. Our results demonstrate that similar pairs potentially regulate different developmental stages depending on the expression values of the corresponding genes. This may define switches between embryonic and maturation programs and could contribute to a better understanding of embryonic cardiac development.

## Author contributions

SZ, CM, and MG participated in the design of the study, conducted computational and statistical analyses. EW supervised the computational and statistical analyses. AR, FR prepared the time course data and the experiments. WZ supervised the experimental design and the experiments. SU prepared and processed the RNAseq data which are used in this study. SZ, CM, RT, and MG were involved in interpretation of the results and the literature survey. MG and SZ wrote the final version of the manuscript. MG conceived of and managed the project. All authors read and approved the final manuscript.

## Funding

SZ was funded by Mediomics (Fördernummer: 01DJ13026B) of the BMBF (German Ministry of Education and Research) and the DZHK. CM was funded by ExiTox (Fördernummer: 031A269C) of the BMBF (German Ministry of Education and Research). WHZ is supported by the DZHK, the German Research Foundation (DFG ZI 708/10-1, SFB 1002 TP C04/S, and SFB 937 A18), the Foundation Leducq, the German Federal Ministry for Science and Education (BMBF FKZ 13GW0007A [BMBF/CIRM ETIII Award]), and the NIH (U01 HL099997).

### Conflict of interest statement

The authors declare that the research was conducted in the absence of any commercial or financial relationships that could be construed as a potential conflict of interest.

## Supplementary material

Supplementary File 1 **DEGList.csv includes all detected stage-specific DEGs**.

Supplementary File 2 **A heatmap of stage-specific DEGs**.

Supplementary File 3 **TFBS pairs found by MatrixCatch for stage-specific DEGs**.

Supplementary File 4 **The stage-specific networks after application of MatrixCatch and Markov clustering algorithm**.

## References

[B1] Akhurst R. J. (2012). The paradoxical TGF-β vasculopathies. Nat. Genet. 44, 838–839. 10.1038/ng.2366 22836090 PMC3543110

[B2] Bergmann O. Bhardwaj R. D. Bernard S. Zdunek S. Barnabé-Heider F. Walsh S.. (2009). Evidence for cardiomyocyte renewal in humans. Science 324, 98–102. 10.1126/science.1164680 19342590 PMC2991140

[B3] Boyer L. A. Lee T. I. Cole M. F. Johnstone S. E. Levine S. S. Zucker J. P.. (2005). Core transcriptional regulatory circuitry in human embryonic stem cells. Cell 122, 947–956. 10.1016/j.cell.2005.08.020 16153702 PMC3006442

[B4] Brade T. Männer J. Kühl M. (2006). The role of Wnt signalling in cardiac development and tissue remodelling in the mature heart. Cardiovasc. Res. 72, 198–209. 10.1016/j.cardiores.2006.06.025 16860783

[B5] Brand T. (2003). Heart development: molecular insights into cardiac specification and early morphogenesis. Dev. Biol. 258, 1–19. 10.1016/S0012-1606(03)00112-X 12781678

[B6] Brewer A. Pizzey J. (2006). GATA factors in vertebrate heart development and disease. Expert. Rev. Mol. Med. 8, 1–20. 10.1017/S1462399406000093 16987437

[B7] Brohée S. van Helden J. (2006). Evaluation of clustering algorithms for protein-protein interaction networks. BMC Bioinformat. 7:488. 10.1186/1471-2105-7-488 17087821 PMC1637120

[B8] Buckingham M. Meilhac S. Zaffran S. (2005). Building the mammalian heart from two sources of myocardial cells. Nat. Rev. Genet. 6, 826–835. 10.1038/nrg1710 16304598

[B9] Burke M. Reisler F. Harrington W. F. (1976). Effect of bridging the two essential thiols of myosin on its spectral and actin-binding properties. Biochemistry 15, 1923–1927. 10.1021/bi00654a020 1268201

[B10] Calvieri C. Rubattu S. Volpe M. (2012). Molecular mechanisms underlying cardiac antihypertrophic and antifibrotic effects of natriuretic peptides. J. Mol. Med. (Berl). 90, 5–13. 10.1007/s00109-011-0801-z 21826523

[B11] Chandra V. Huang P. Potluri N. Wu D. Kim Y. Rastinejad F. (2013). Multidomain integration in the structure of the HNF-4α nuclear receptor complex. Nature 495, 394–398. 10.1038/nature11966 23485969 PMC3606643

[B12] Chaudhry B. Ramsbottom S. Henderson D. J. (2014). Genetics of cardiovascular development. Prog. Mol. Biol. Transl. Sci. 124, 19–41. 10.1016/B978-0-12-386930-2.00002-1 24751425

[B13] Chen B. P. Liang G. Whelan J. Hai T. (1994). ATF3 and ATF3Δ zip. Transcriptional repression versus activation by alternatively spliced isoforms. J. Biol. Chem. 269, 15819–15826. 7515060

[B14] Chen X. Shevtsov S. P. Hsich E. Cui L. Haq S. Aronovitz M.. (2006). The β-catenin/T-cell factor/lymphocyte enhancer factor signaling pathway is required for normal and stress-induced cardiac hypertrophy. Mol. Cell. Biol. 26, 4462–4473. 10.1128/MCB.02157-05 16738313 PMC1489123

[B15] Chou B.-K. Mali P. Huang X. Ye Z. Dowey S. N. Resar L. M.. (2011). Efficient human iPS cell derivation by a non-integrating plasmid from blood cells with unique epigenetic and gene expression signatures. Cell. Res. 21, 518–529. 10.1038/cr.2011.12 21243013 PMC3193421

[B16] Crabtree G. R. Olson E. N. (2002). NFAT signaling: choreographing the social lives of cells. Cell 109 (Suppl.), S67–S79. 10.1016/s0092-8674(02)00699-2 11983154

[B17] Dewey F. E. Perez M. V. Wheeler M. T. Watt C. Spin J. Langfelder P.. (2011). Gene coexpression network topology of cardiac development, hypertrophy, and failure. Circ. Cardiovasc. Genet. 4, 26–35. 10.1161/CIRCGENETICS.110.941757 21127201 PMC3324316

[B18] Deyneko I. V. Kel A. E. Kel-Margoulis O. V. Deineko E. V. Wingender E. Weiss S. (2013). MatrixCatch–a novel tool for the recognition of composite regulatory elements in promoters. BMC Bioinformat. 14:241. 10.1186/1471-2105-14-241 23924163 PMC3754795

[B19] Didié M. Christalla P. Rubart M. Muppala V. Döker S. Unsöld B.. (2013). Parthenogenetic stem cells for tissue-engineered heart repair. J. Clin. Invest. 123, 1285–1298. 10.1172/JCI66854 23434590 PMC3582145

[B20] Domazet-Lošo T. Tautz D. (2010). A phylogenetically based transcriptome age index mirrors ontogenetic divergence patterns. Nature 468, 815–818. 10.1038/nature09632 21150997

[B21] Dongen S. (2000). Graph Clustering by Flow Simulation. PhD thesis, University of Utrecht, Netherlands.

[B22] Duboule D. (1994). Temporal colinearity and the phylotypic progression: a basis for the stability of a vertebrate Bauplan and the evolution of morphologies through heterochrony. Dev. Suppl. 135–142. Available online at: http://dev.biologists.org/content/develop/1994/Supplement/135.full.pdf 7579514

[B23] Dyer L. A. Pi X. Patterson C. (2014). The role of BMPs in endothelial cell function and dysfunction. Trends Endocrinol. Metab. 25, 472–480. 10.1016/j.tem.2014.05.003 24908616 PMC4149816

[B24] Euler G. (2015). Good and bad sides of TGFβ-signaling in myocardial infarction. Front. Physiol. 6:66. 10.3389/fphys.2015.00066 25788886 PMC4349055

[B25] Euler-Taimor G. Heger J. (2006). The complex pattern of SMAD signaling in the cardiovascular system. Cardiovasc Res. 69, 15–25. 10.1016/j.cardiores.2005.07.007 16107248

[B26] Fortin J. Ongaro L. Li Y. Tran S. Lamba P. Wang Y.. (2015). Minireview: Activin signaling in gonadotropes: What does the FOX say to the SMAD? Mol. Endocrinol. 29, 963–977. 10.1210/me.2015-1004 25942106 PMC5414707

[B27] Fusco A. Fedele M. (2007). Roles of HMGA proteins in cancer. Nat. Rev. Cancer 7, 899–910. 10.1038/nrc2271 18004397

[B28] Gao Z. Kim G. H. Mackinnon A. C. Flagg A. E. Bassett B. Earley J. U.. (2010). Ets1 is required for proper migration and differentiation of the cardiac neural crest. Development 137, 1543–1551. 10.1242/dev.047696 20356956 PMC2853851

[B29] Gilchrist M. Thorsson V. Li B. Rust A. G. Korb M. Roach J. C.. (2006). Systems biology approaches identify ATF3 as a negative regulator of Toll-like receptor 4. Nature 441, 173–178. 10.1038/nature04768 16688168

[B30] Gordon J. W. Shaw J. A. Kirshenbaum L. A. (2011). Multiple facets of NF-κB in the heart: to be or not to NF-κB. Circ. Res. 108, 1122–1132. 10.1161/CIRCRESAHA.110.226928 21527742

[B31] Graef I. A. Chen F. Crabtree G. R. (2001). NFAT signaling in vertebrate development. Curr. Opin. Genet. Dev. 11, 505–512. 10.1016/S0959-437X(00)00225-2 11532391

[B32] Guo Y. Costa R. Ramsey H. Starnes T. Vance G. Robertson K.. (2002). The embryonic stem cell transcription factors Oct-4 and FoxD3 interact to regulate endodermal-specific promoter expression. Proc. Natl. Acad. Sci. U.S.A. 99, 3663–3667. 10.1073/pnas.062041099 11891324 PMC122580

[B33] Heldin C. H. Miyazono K. ten Dijke P. (1997). TGF-β signalling from cell membrane to nucleus through SMAD proteins. Nature 390, 465–471. 10.1038/37284 9393997

[B34] Hermann-Kleiter N. Baier G. (2010). NFAT pulls the strings during CD4+ T helper cell effector functions. Blood 115, 2989–2997. 10.1182/ablood-2009-10-233585 20103781

[B35] Herzig T. C. Jobe S. M. Aoki H. Molkentin J. D. Cowley A. W. Jr Izumo S.. (1997). Angiotensin II type1a receptor gene expression in the heart: AP-1 and GATA-4 participate in the response to pressure overload. Proc. Natl. Acad. Sci. U.S.A. 94, 7543–7548. 10.1073/pnas.94.14.7543 9207128 PMC23858

[B36] Hess J. Angel P. Schorpp-Kistner M. (2004). AP-1 subunits: quarrel and harmony among siblings. J. Cell Sci. 117(Pt 25), 5965–5973. 10.1242/jcs.01589 15564374

[B37] Hillion J. Dhara S. Sumter T. F. Mukherjee M. Di Cello F. Belton A.. (2008). The high-mobility group A1a/signal transducer and activator of transcription-3 axis: an achilles heel for hematopoietic malignancies? Cancer Res. 68, 10121–10127. 10.1158/0008-5472.can-08-2121 19074878 PMC2913892

[B38] Hillion J. Wood L. J. Mukherjee M. Bhattacharya R. Di Cello F. Kowalski J.. (2009). Upregulation of MMP-2 by HMGA1 promotes transformation in undifferentiated, large-cell lung cancer. Mol. Cancer Res. 7, 1803–1812. 10.1158/1541-7786.MCR-08-0336 19903768 PMC3069640

[B39] Himes S. R. Coles L. S. Reeves R. Shannon M. F. (1996). High mobility group protein I(Y) is required for function and for c-Rel binding to CD28 response elements within the GM-CSF and IL-2 promoters. Immunity 5, 479–489. 8934574 10.1016/s1074-7613(00)80503-8

[B40] Hogan P. G. Chen L. Nardone J. Rao A. (2003). Transcriptional regulation by calcium, calcineurin, and NFAT. Genes Dev. 17, 2205–2232. 10.1101/gad.1102703 12975316

[B41] Hu Z. Gallo S. M. (2010). Identification of interacting transcription factors regulating tissue gene expression in human. BMC Genomics 11:49. 10.1186/1471-2164-11-49 20085649 PMC2822763

[B42] Ishiguro T. Nagawa H. Naito M. Tsuruo T. (2000). Inhibitory effect of ATF3 antisense oligonucleotide on ectopic growth of HT29 human colon cancer cells. Jpn. J. Cancer Res. 91, 833–836. 10.1111/j.1349-7006.2000.tb01021.x 10965025 PMC5926425

[B43] Jolma A. Yan J. Whitington T. Toivonen J. Nitta K. R. Rastas P.. (2013). DNA-Binding Specificities of Human Transcription Factors. Cell 152, 327–339. 10.1016/j.cell.2012.12.009 23332764

[B44] Jolma A. Yin Y. Nitta K. R. Dave K. Popov A. Taipale M.. (2015). DNA-dependent formation of transcription factor pairs alters their binding specificity. Nature 527, 384–388. 10.1038/nature15518 26550823

[B45] Kalinka A. T. Varga K. M. Gerrard D. T. Preibisch S. Corcoran D. L. Jarrells J.. (2010). Gene expression divergence recapitulates the developmental hourglass model. Nature 468, 811–814. 10.1038/nature09634 21150996

[B46] Karolchik D. Hinrichs A. S. Furey T. S. Roskin K. M. Sugnet C. W. Haussler D.. (2004). The UCSC Table Browser data retrieval tool. Nucleic Acids Res. 32(Suppl. 1), D493–D496. 10.1093/nar/gkh103 14681465 PMC308837

[B47] Kel-Margoulis O. V. Kel A. E. Reuter I. Deineko I. V. Wingender E. (2002). TRANSCompel: a database on composite regulatory elements in eukaryotic genes. Nucleic Acids Res. 30, 332–334. 10.1093/nar/30.1.332 11752329 PMC99108

[B48] Khan A. Hyde R. K. Dutra A. Mohide P. Liu P. (2006). Core binding factor beta (CBFB) haploinsufficiency due to an interstitial deletion at 16q21q22 resulting in delayed cranial ossification, cleft palate, congenital heart anomalies, and feeding difficulties but favorable outcome. Am. J. Med. Genet. A 140, 2349–2354. 10.1002/ajmg.a.31479 17022082

[B49] Kirby M. L. (2002). Molecular embryogenesis of the heart. Pediatr. Dev. Pathol. 5, 516–543. 10.1007/s10024-002-0004-2 12297889

[B50] Kwon C. Arnold J. Hsiao E. C. Taketo M. M. Conklin B. R. Srivastava D. (2007). Canonical Wnt signaling is a positive regulator of mammalian cardiac progenitors. Proc. Natl. Acad. Sci. U.S.A. 104, 10894–10899. 10.1073/pnas.0704044104 17576928 PMC1904134

[B51] Leask A. (2007). TGFβ, cardiac fibroblasts, and the fibrotic response. Cardiovasc Res. 74, 207–212. 10.1016/j.cardiores.2006.07.012 16919613

[B52] Leask A. Abraham D. J. (2004). TGF-β signaling and the fibrotic response. FASEB J. 18, 816–827. 10.1096/fj.03-1273rev 15117886

[B53] Lewis H. Kaszubska W. DeLamarter J. F. Whelan J. (1994). Cooperativity between two NF-κB complexes, mediated by high-mobility-group protein I(Y), is essential for cytokine-induced expression of the E-selectin promoter. Mol. Cell Biol. 14, 5701–5709. 10.1128/MCB.14.9.5701 7520524 PMC359095

[B54] Lin H. Li H.-F. Chen H.-H. Lai P.-F. Juan S.-H. Chen J.-J.. (2014). Activating transcription factor 3 protects against pressure-overload heart failure via the autophagy molecule Beclin-1 pathway. Mol. Pharmacol. 85, 682–691. 10.1124/mol.113.090092 24550138

[B55] Linnemann A. K. O'Geen H. Keles S. Farnham P. J. Bresnick E. H. (2011). Genetic framework for GATA factor function in vascular biology. Proc. Natl. Acad. Sci. U.S.A. 108, 13641–13646. 10.1073/pnas.1108440108 21808000 PMC3158141

[B56] Liu Q. Chen Y. Auger-Messier M. Molkentin J. D. (2012). Interaction between NFκB and NFAT coordinates cardiac hypertrophy and pathological remodeling. Circ. Res. 110, 1077–1086. 10.1161/CIRCRESAHA.111.260729 22403241 PMC3341669

[B57] Macián F. (2005). NFAT proteins: key regulators of T-cell development and function. Nat. Rev. Immunol. 5, 472–484. 10.1038/nri1632 15928679

[B58] Macián F. López-Rodríguez C. Rao A. (2001). Partners in transcription: NFAT and AP-1. Oncogene 20, 2476–2489. 10.1038/sj.onc.1204386 11402342

[B59] Macias M. J. Martin-Malpartida P. Massagué J. (2015). Structural determinants of Smad function in TGF-β signaling. Trends Biochem. Sci. 40, 296–308. 10.1016/j.tibs.2015.03.012 25935112 PMC4485443

[B60] Maeda J. Yamagishi H. McAnally J. Yamagishi C. Srivastava D. (2006). Tbx1 is regulated by forkhead proteins in the secondary heart field. Dev. Dyn. 235, 701–710. 10.1002/dvdy.20686 16444712 PMC3316489

[B61] Mantovani F. Covaceuszach S. Rustighi A. Sgarra R. Heath C. Goodwin G. H.. (1998). NF-κB mediated transcriptional activation is enhanced by the architectural factor HMGI-C. Nucleic Acids Res. 26, 1433–1439. 10.1093/nar/26.6.1433 9490789 PMC147413

[B62] Martin J. Afouda B. A. Hoppler S. (2010). Wnt/β-catenin signalling regulates cardiomyogenesis via GATA transcription factors. J. Anat. 216, 92–107. 10.1111/j.1469-7580.2009.01171.x 20402826 PMC2807978

[B63] Martin L. J. Bergeron F. Viger R. S. Tremblay J. J. (2012). Functional cooperation between GATA factors and cJUN on the star promoter in MA-10 leydig cells. J. Androl. 33, 81–87. 10.2164/jandrol.110.012039 21350237

[B64] Massagué J. (2012). TGFβ signalling in context. Nat. Rev. Mol. Cell Biol. 13, 616–630. 10.1038/nrm3434 22992590 PMC4027049

[B65] Mayr B. Montminy M. (2001). Transcriptional regulation by the phosphorylation-dependent factor CREB. Nat. Rev. Mol. Cell. Biol. 2, 599–609. 10.1038/35085068 11483993

[B66] Meckbach C. Tacke R. Hua X. Waack S. Wingender E. Gültas M. (2015). PC-TraFF: identification of potentially collaborating transcription factors using pointwise mutual information. BMC Bioinformat. 16:400. 10.1186/s12859-015-0827-2 26627005 PMC4667426

[B67] Miura G. I. Yelon D. (2013). Cardiovascular biology: play it again, Gata4. Curr. Biol. 23, R619–R621. 10.1016/j.cub.2013.06.006 23885880

[B68] Naito A. T. Shiojima I. Komuro I. (2010). Wnt signaling and aging-related heart disorders. Circ. Res. 107, 1295–1303. 10.1161/CIRCRESAHA.110.223776 21106946

[B69] Neph S. Stergachis A. B. Reynolds A. Sandstrom R. Borenstein E. Stamatoyannopoulos J. A. (2012). Circuitry and dynamics of human transcription factor regulatory networks. Cell 150, 1274–1286. 10.1016/j.cell.2012.04.040 22959076 PMC3679407

[B70] Nichols J. Zevnik B. Anastassiadis K. Niwa H. Klewe-Nebenius D. Chambers I.. (1998). Formation of pluripotent stem cells in the mammalian embryo depends on the POU transcription factor Oct4. Cell 95, 379–391. 10.1016/S0092-8674(00)81769-9 9814708

[B71] Odom D. T. Dowell R. D. Jacobsen E. S. Nekludova L. Rolfe P. A. Danford T. W.. (2006). Core transcriptional regulatory circuitry in human hepatocytes. Mol. Syst. Biol. 2:2006.0017. 10.1038/msb4100059 16738562 PMC1681491

[B72] Ohneda K. Yamamoto M. (2002). Roles of hematopoietic transcription factors GATA-1 and GATA-2 in the development of red blood cell lineage. Acta. Haematol. 108, 237–245. 10.1159/000065660 12432220

[B73] Orkin S. H. (1992). GATA-binding transcription factors in hematopoietic cells. Blood 80, 575–581. 1638017

[B74] Pal R. Khanna A. (2006). Role of Smad- and Wnt-dependent pathways in embryonic cardiac development. Stem Cells Dev. 15, 29–39. 10.1089/scd.2006.15.29 16522160

[B75] Perrella M. A. Pellacani A. Wiesel P. Chin M. T. Foster L. C. Ibanez M.. (1999). High mobility group-I(Y) protein facilitates nuclear factor-κB binding and transactivation of the inducible nitric-oxide synthase promoter/enhancer. J. Biol. Chem. 274, 9045–9052. 10085153 10.1074/jbc.274.13.9045

[B76] Pesce M. Schöler H. R. (2001). Oct-4: gatekeeper in the beginnings of mammalian development. Stem Cells 19, 271–278. 10.1634/stemcells.19-4-271 11463946

[B77] Peterkin T. Gibson A. Loose M. Patient R. (2005). The roles of GATA-4, -5 and -6 in vertebrate heart development. Semin Cell Dev. Biol. 16, 83–94. 10.1016/j.semcdb.2004.10.003 15659343

[B78] Pikkarainen S. Tokola H. Kerkelä R. Ruskoaho H. (2004). GATA transcription factors in the developing and adult heart. Cardiovasc Res. 63, 196–207. 10.1016/j.cardiores.2004.03.025 15249177

[B79] Quint M. Drost H.-G. Gabel A. Ullrich K. K. Bönn M. Grosse I. (2012). A transcriptomic hourglass in plant embryogenesis. Nature 490, 98–101. 10.1038/nature11394 22951968

[B80] R Core Team (2015). R: A Language and Environment for Statistical Computing. Vienna: R Foundation for Statistical Computing.

[B81] Raff R. A. Wolpert L. (1996). The shape of life-genes, development and evolution of animal forms. Genet. Res. 68:261.

[B82] Resar L. M. S. (2010). The high mobility group A1 gene: transforming inflammatory signals into cancer? Cancer Res. 70, 436–439. 10.1158/0008-5472.can-09-1212 20068164 PMC2807996

[B83] Ruiz-Ortega M. Rodríguez-Vita J. Sanchez-Lopez E. Carvajal G. Egido J. (2007). TGF-β signaling in vascular fibrosis. Cardiovasc. Res. 74, 196–206. 10.1016/j.cardiores.2007.02.008 17376414

[B84] Ryan K. Chin A. J. (2003). T-box genes and cardiac development. Birth Defects Res. C Embryo Today 69, 25–37. 10.1002/bdrc.10001 12768655

[B85] Schleich J.-M. Abdulla T. Summers R. Houyel L. (2013). An overview of cardiac morphogenesis. Arch. Cardiovasc. Dis. 106, 612–623. 10.1016/j.acvd.2013.07.001 24138816

[B86] Schneiders D. Heger J. Best P. Piper H. M. Taimor G. (2005). SMAD proteins are involved in apoptosis induction in ventricular cardiomyocytes. Cardiovasc. Res. 67, 87–96. 10.1016/j.cardiores.2005.02.021 15949472

[B87] Schöler H. R. Ruppert S. Suzuki N. Chowdhury K. Gruss P. (1990). New type of POU domain in germ line-specific protein Oct-4. Nature 344, 435–439. 1690859 10.1038/344435a0

[B88] Schröder D. Heger J. Piper H. M. Euler G. (2006). Angiotensin II stimulates apoptosis via TGF-β1 signaling in ventricular cardiomyocytes of rat. J. Mol. Med. (Berl). 84, 975–983. 10.1007/s00109-006-0090-0 16924465

[B89] Schubert W. Yang X. Y. Yang T. T. C. Factor S. M. Lisanti M. P. Molkentin J. D.. (2003). Requirement of transcription factor NFAT in developing atrial myocardium. J. Cell. Biol. 161, 861–874. 10.1083/jcb.200301058 12796475 PMC2172977

[B90] Schuldenfrei A. Belton A. Kowalski J. Talbot C. C. Jr, Di Cello, F. Poh W.. (2011). HMGA1 drives stem cell, inflammatory pathway, and cell cycle progression genes during lymphoid tumorigenesis. BMC Genomics 12:549. 10.1186/1471-2164-12-549 22053823 PMC3245506

[B91] Schulz R. A. Yutzey K. E. (2004). Calcineurin signaling and NFAT activation in cardiovascular and skeletal muscle development. Dev. Biol. 266, 1–16. 10.1016/j.ydbio.2003.10.008 14729474

[B92] Seo S. Kume T. (2006). Forkhead transcription factors, Foxc1 and Foxc2, are required for the morphogenesis of the cardiac outflow tract. Dev. Biol. 296, 421–436. 10.1016/j.ydbio.2006.06.012 16839542

[B93] Shah S. N. Kerr C. Cope L. Zambidis E. Liu C. Hillion J.. (2012). HMGA1 reprograms somatic cells into pluripotent stem cells by inducing stem cell transcriptional networks. PLoS ONE 7:e48533. 10.1371/journal.pone.0048533 23166588 PMC3499526

[B94] Shaulian E. (2010). AP-1–The Jun proteins: Oncogenes or tumor suppressors in disguise? Cell Signal. 22, 894–899. 10.1016/j.cellsig.2009.12.008 20060892

[B95] Shaulian E. Karin M. (2002). AP-1 as a regulator of cell life and death. Nat. Cell. Biol. 4, E131–E136. 10.1038/ncb0502-e131 11988758

[B96] Shi G. Jin Y. (2010). Role of Oct4 in maintaining and regaining stem cell pluripotency. Stem Cell Res. Ther. 1:39. 10.1186/scrt39 21156086 PMC3025441

[B97] Shih Y.-K. Parthasarathy S. (2012). Identifying functional modules in interaction networks through overlapping Markov clustering. Bioinformatics 28, i473–i479. 10.1093/bioinformatics/bts370 22962469 PMC3436797

[B98] Smyth G. K. (2004), Linear models empirical bayes methods for assessing differential expression in microarray experiments. Stat. Appl. Genet. Mol. Biol. 3, 1–25. 10.2202/1544-6115.1027. 16646809

[B99] Soong P. L. Tiburcy M. Zimmermann W.-H. (2012). Cardiac differentiation of human embryonic stem cells and their assembly into engineered heart muscle. Curr. Protoc. Cell Biol. Chapter 23:Unit23.8. 10.1002/0471143030.cb2308s55 23129117

[B100] Suzuki Y. J. Ikeda T. Shi S. S. Kitta K. Kobayashi Y. M. Morad M.. (1999). Regulation of GATA-4 and AP-1 in transgenic mice overexpressing cardiac calsequestrin. Cell Calcium 25, 401–407. 10579051 10.1054/ceca.1999.0037

[B101] Sylva M. van den Hoff M. J. B. Moorman A. F. M. (2014). Development of the human heart. Am. J. Med. Genet. A 164A, 1347–1371. 10.1002/ajmg.a.35896 23633400

[B102] Takeuchi T. (2014). Regulation of cardiomyocyte proliferation during development and regeneration. Dev. Growth. Differ. 56, 402–409. 10.1111/dgd.12134 24738847

[B103] Thanos D. Maniatis T. (1992). The high mobility group protein HMG I(Y) is required for NF-κB-dependent virus induction of the human IFN-β gene. Cell 71, 777–789. 1330326 10.1016/0092-8674(92)90554-p

[B104] Thanos D. Maniatis T. (1996). In vitro assembly of enhancer complexes. Methods Enzymol 274, 162–173. 8902803 10.1016/s0076-6879(96)74015-6

[B105] Tiburcy M. Zimmermann W.-H. (2014). Modeling myocardial growth and hypertrophy in engineered heart muscle. Trends Cardiovasc. Med. 24, 7–13. 10.1016/j.tcm.2013.05.003 23953977

[B106] Turbendian H. K. Gordillo M. Tsai S.-Y. Lu J. Kang G. Liu T.-C.. (2013). GATA factors efficiently direct cardiac fate from embryonic stem cells. Development 140, 1639–1644. 10.1242/dev.093260 23487308 PMC3621482

[B107] Vlasblom J. Wodak S. J. (2009). Markov clustering versus affinity propagation for the partitioning of protein interaction graphs. BMC Bioinformatics 10:99. 10.1186/1471-2105-10-99 19331680 PMC2682798

[B108] Wang X. Jauch R. (2014). OCT4: A penetrant pluripotency inducer. Cell Regen (Lond). 3:6. 10.1186/2045-9769-3-6 25408885 PMC4230516

[B109] Watt A. J. Garrison W. D. Duncan S. A. (2003). HNF4: a central regulator of hepatocyte differentiation and function. Hepatology 37, 1249–1253. 10.1053/jhep.2003.50273 12774000

[B110] Whitfield T. W. Wang J. Collins P. J. Partridge E. C. Aldred S. F. Trinklein N. D.. (2012). Functional analysis of transcription factor binding sites in human promoters. Genome Biol. 13:R50. 10.1186/gb-2012-13-9-r50 22951020 PMC3491394

[B111] Williams M. D. Zhang X. Belton A. S. Xian L. Huso T. Park J.-J.. (2015). HMGA1 drives metabolic reprogramming of intestinal epithelium during hyperproliferation, polyposis, and colorectal carcinogenesis. J. Proteome Res. 14, 1420–1431. 10.1021/pr501084s 25643065

[B112] Wong K.-C. Li Y. Peng C. (2016). Identification of coupling DNA motif pairs on long-range chromatin interactions in human K562 cells. Bioinformatics 32, 321–324. 10.1093/bioinformatics/btv555 26411866

[B113] Wood L. D. Farmer A. A. Richmond A. (1995). HMGI(Y) and Sp1 in addition to NF-κB regulate transcription of the MGSA/GROα gene. Nucleic Acids Res. 23, 4210–4219. 10.1093/nar/23.20.4210 7479086 PMC307364

[B114] Yamagishi H. Maeda J. Hu T. McAnally J. Conway S. J. Kume T.. (2003). Tbx1 is regulated by tissue-specific forkhead proteins through a common Sonic hedgehog-responsive enhancer. Genes Dev. 17, 269–281. 10.1101/gad.1048903 12533514 PMC195981

[B115] Yan C. Lu D. Hai T. Boyd D. D. (2005). Activating transcription factor 3, a stress sensor, activates p53 by blocking its ubiquitination. EMBO J. 24, 2425–2435. 10.1038/sj.emboj.7600712 15933712 PMC1173153

[B116] Yang T. T. C. Chow C.-W. (2003). Transcription cooperation by NFAT.C/EBP composite enhancer complex. J. Biol. Chem. 278, 15874–15885. 10.1074/jbc.M211560200 12606546

[B117] Ye L. Zimmermann W.-H. Garry D. J. Zhang J. (2013). Patching the heart: cardiac repair from within and outside. Circ. Res. 113, 922–932. 10.1161/CIRCRESAHA.113.300216 24030022 PMC3886802

[B118] Yin X. Wolford C. C. Chang Y.-S. McConoughey S. J. Ramsey S. A. Aderem A.. (2010). ATF3, an adaptive-response gene, enhances TGFβ signaling and cancer-initiating cell features in breast cancer cells. J. Cell Sci. 123(Pt 20), 3558–3565. 10.1242/jcs.064915 20930144 PMC2951469

[B119] Yuan H.-F. Huang H. Li X.-Y. Guo W. Xing W. Sun Z.-Y.. (2013). A dual AP-1 and SMAD decoy ODN suppresses tissue fibrosis and scarring in mice. J. Invest. Dermatol. 133, 1080–1087. 10.1038/jid.2012.443 23223130

[B120] Zhang X. M. Verdine G. L. (1999). A small region in HMG I(Y) is critical for cooperation with NF-κB on DNA. J. Biol. Chem. 274, 20235–20243. 10.1074/jbc.274.29.20235 10400641

[B121] Zhou H. Yang H.-X. Yuan Y. Deng W. Zhang J.-Y. Bian Z.-Y.. (2013). Paeoniflorin attenuates pressure overload-induced cardiac remodeling via inhibition of TGFβ/Smads and NF-κB pathways. J. Mol. Histol. 44, 357–367. 10.1007/s10735-013-9491-x 23417833

[B122] Zimmermann W.-H. Melnychenko I. Wasmeier G. Didié M. Naito H. Nixdorff U.. (2006). Engineered heart tissue grafts improve systolic and diastolic function in infarcted rat hearts. Nat. Med. 12, 452–458. 10.1038/nm1394 16582915

[B123] Zobalova R. Swettenham E. Chladova J. Dong L.-F. Neuzil J. (2008). Daxx inhibits stress-induced apoptosis in cardiac myocytes. Redox. Rep. 13, 263–270. 10.1179/135100008X308975 19017466

