# Supplementary figures and images for: Computational Detection of Stage-Specific Transcription Factor Clusters during Heart Development
Source: Front Genet. 2016 Mar 23;7:33. doi: 10.3389/fgene.2016.00033 (PMC4804722; doi:10.3389/fgene.2016.00033)

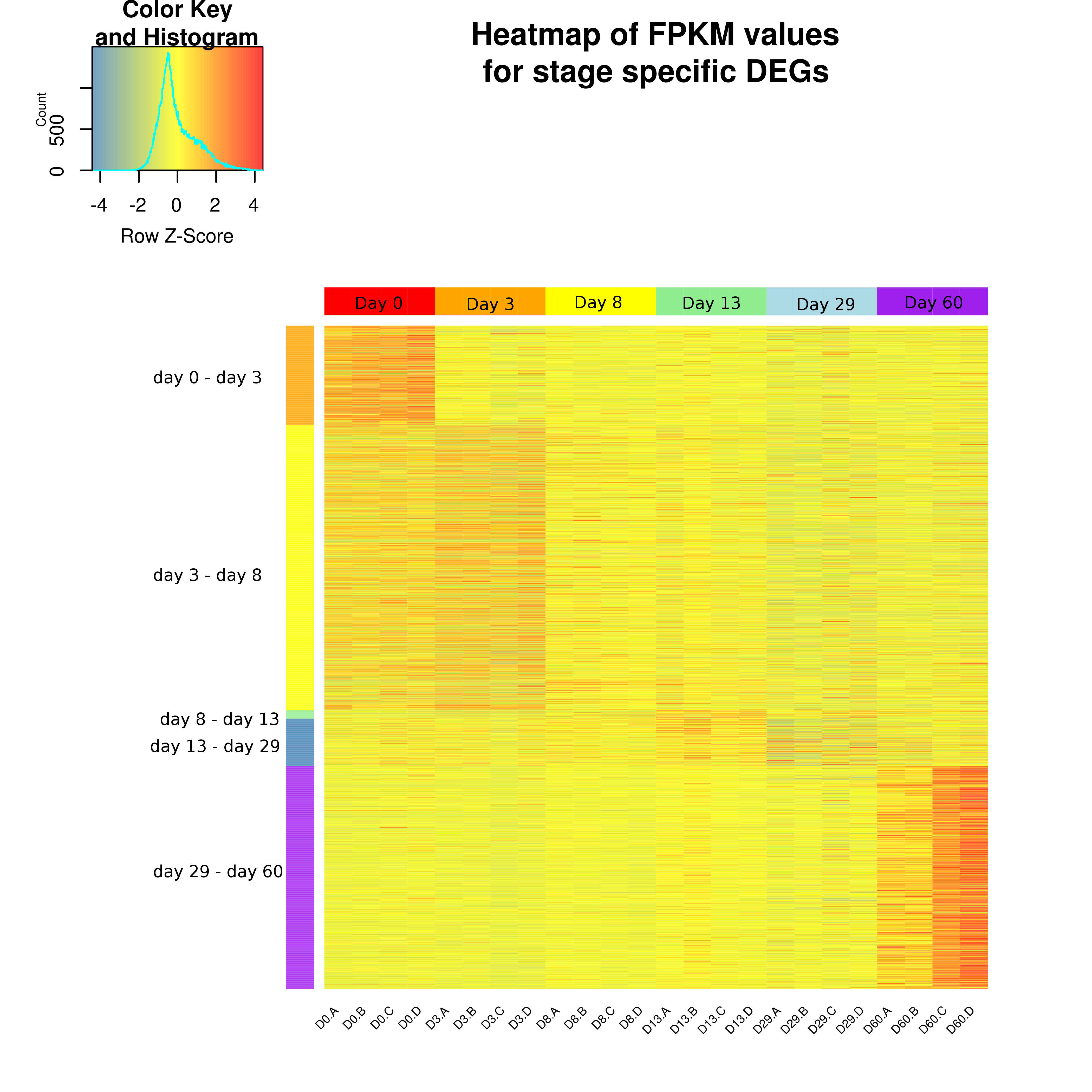

Supplement: Supplementary File 2 — A heatmap of stage-specific DEGs. [file Image1.jpg]
