# Supplementary material for: Computational Detection of Stage-Specific Transcription Factor Clusters during Heart Development
Source: Front Genet. 2016 Mar 23;7:33. doi: 10.3389/fgene.2016.00033 (PMC4804722; doi:10.3389/fgene.2016.00033)
Supplement: Supplementary File 4 — The stage-specific networks after application of MatrixCatch and Markov clustering algorithm. [file Presentation1.PDF]

# *Supplementary Material*: Computational detection of stage-specific transcription factor clusters during heart development

**Sebastian Zeidler [1,2,4*], Cornelia Meckbach[1], Rebecca Tacke[1], Farah S. Raad[2,4], Angelica Roa[2,4], Shizuka Uchida[3,5], Wolfram-Hubertus Zimmermann[2,4], Edgar Wingender[1,4] and Mehmet Gültas[1]**

[1] *Institute of Bioinformatics, University Medical Center Göttingen, Georg-August-University Göttingen, Goldschmidtstraße 1, 37077 Göttingen, Germany*
[2] *Institute of Pharmacology and Toxicology, Heart Research Center Göttingen, University Medical Center Göttingen, Georg-August-University Göttingen, Robert-Koch-Str. 40, 37075 Göttingen, Germany*
[3] *Institute of Cardiovascular Regeneration, Goethe University Frankfurt, Theodor-Stern-Kai 7, 60590 Frankfurt am Main, Germany*
[4] *DZHK (German Centre for Cardiovascular Research), Partner site Göttingen, Göttingen, Germany*
[5] *DZHK (German Centre for Cardiovascular Research), Partner site Rhein-Main, Frankfurt am Main, Germany*

Correspondence*:
Sebastian Zeidler
Institute of Bioinformatics, University Medical Center Göttingen, Goldschmidtstraße 1, 37077 Göttingen, Germany, sebastian.zeidler@bioinf.med.uni-goettingen.de

## 1 SUPPLEMENTARY FILE 4

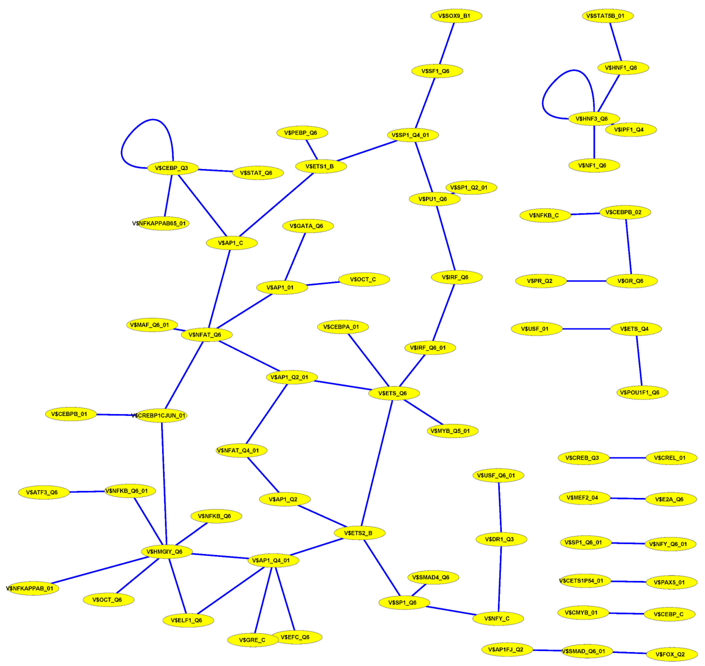

**Supplementary Figure 1.** Identified MatrixCatch pairs at day 0 - day 3 (mesoderm induction stage).

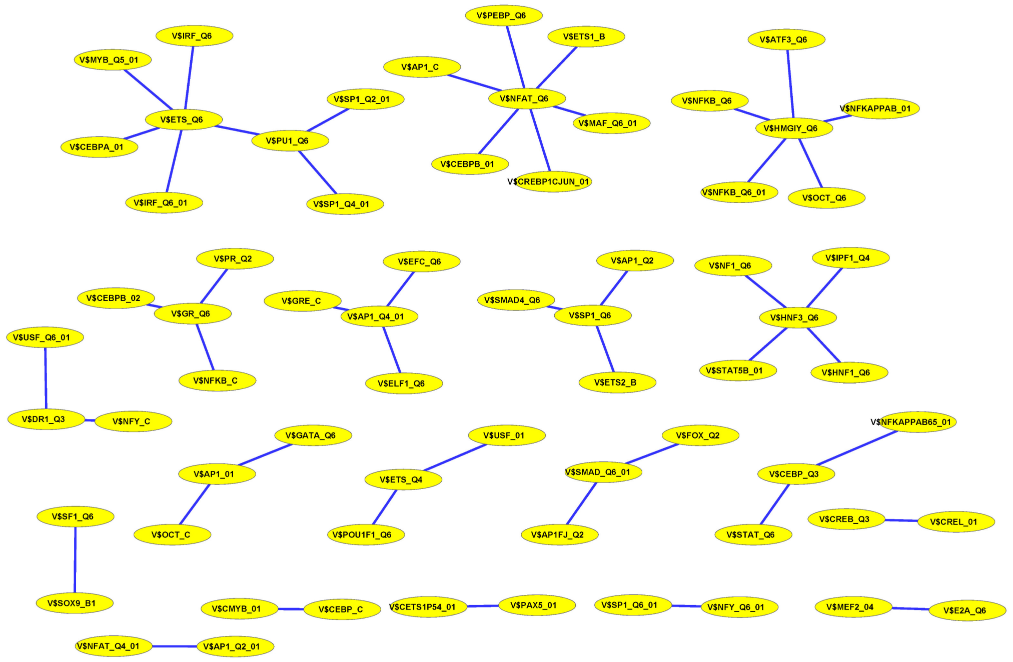


**Supplementary Figure 2.** Identified MatrixCatch pairs after utilization of the Markov clustering algorithm for day 0 - day 3 (mesoderm induction stage).

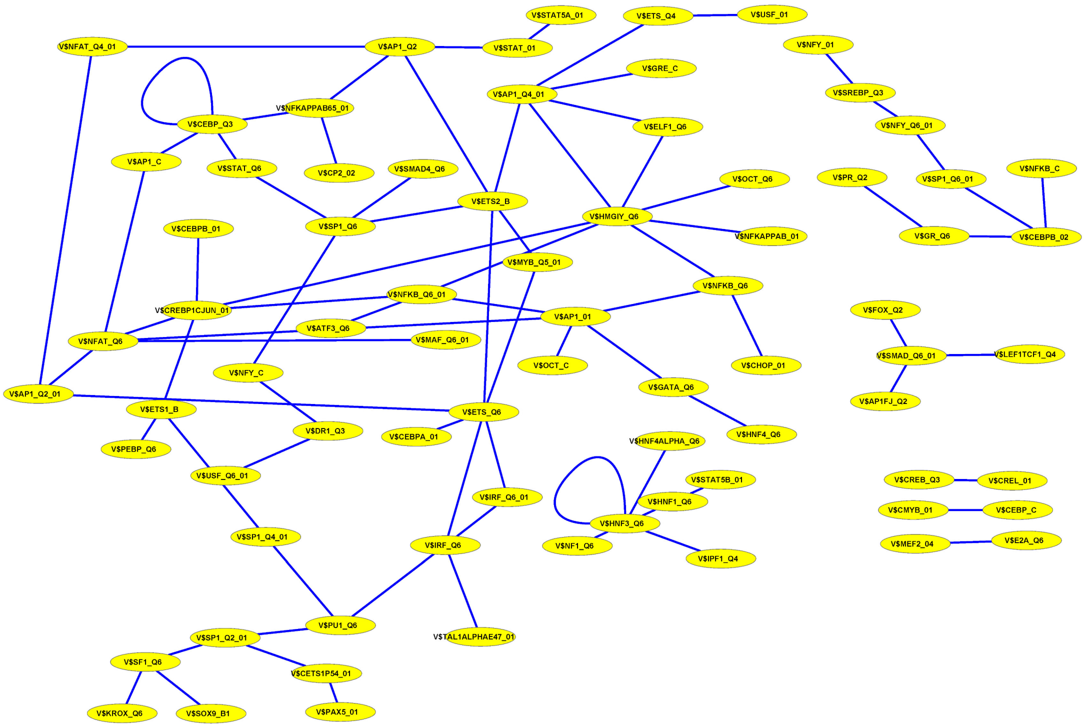


**Supplementary Figure 3.** Identified MatrixCatch pairs at day 3 - day 8 (early cardiac specification stage).

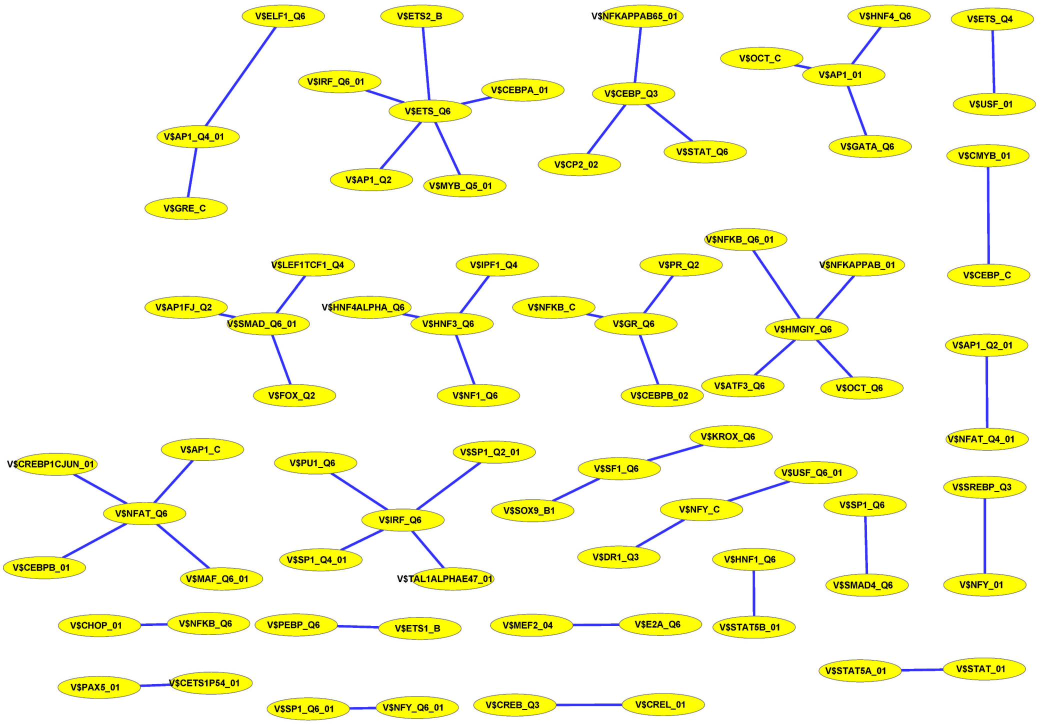


**Supplementary Figure 4.** Identified MatrixCatch pairs after utilization of the Markov clustering algorithm for day 3 - day 8 (early cardiac specification stage).

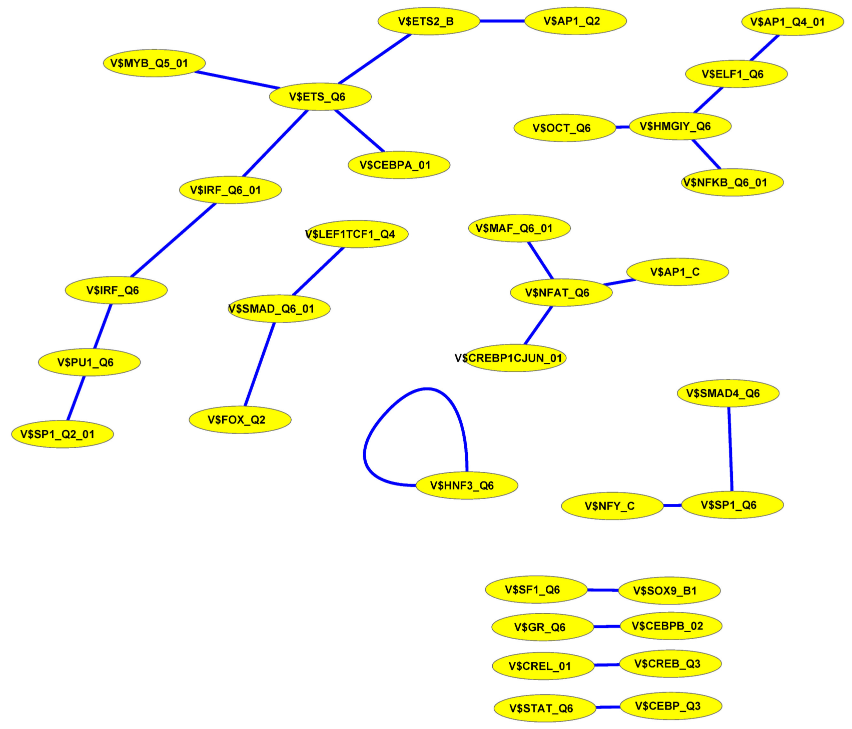


**Supplementary Figure 5.** Identified MatrixCatch pairs at day 8 - day 13 (late cardiac specification stage).

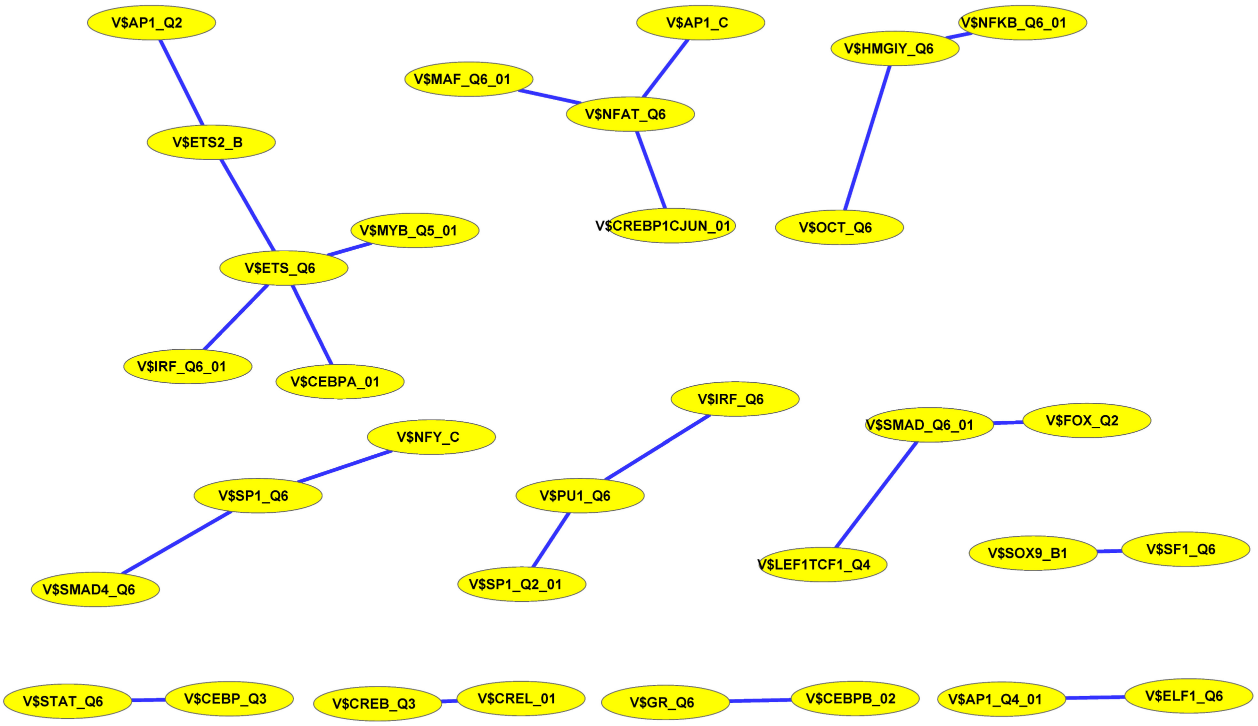


**Supplementary Figure 6.** Identified MatrixCatch pairs after utilization of the Markov clustering algorithm for day 8 - day 13 (late cardiac specification stage).

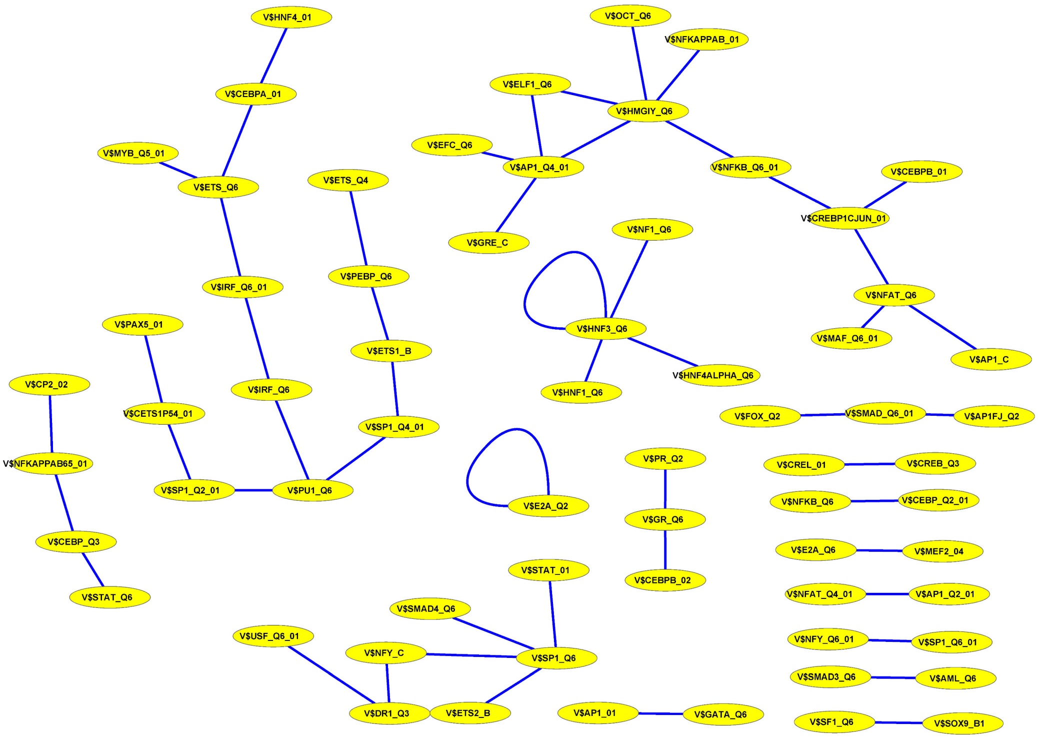


**Supplementary Figure 7.** Identified MatrixCatch pairs at day 13 - day 29 (early cardiac maturation stage).

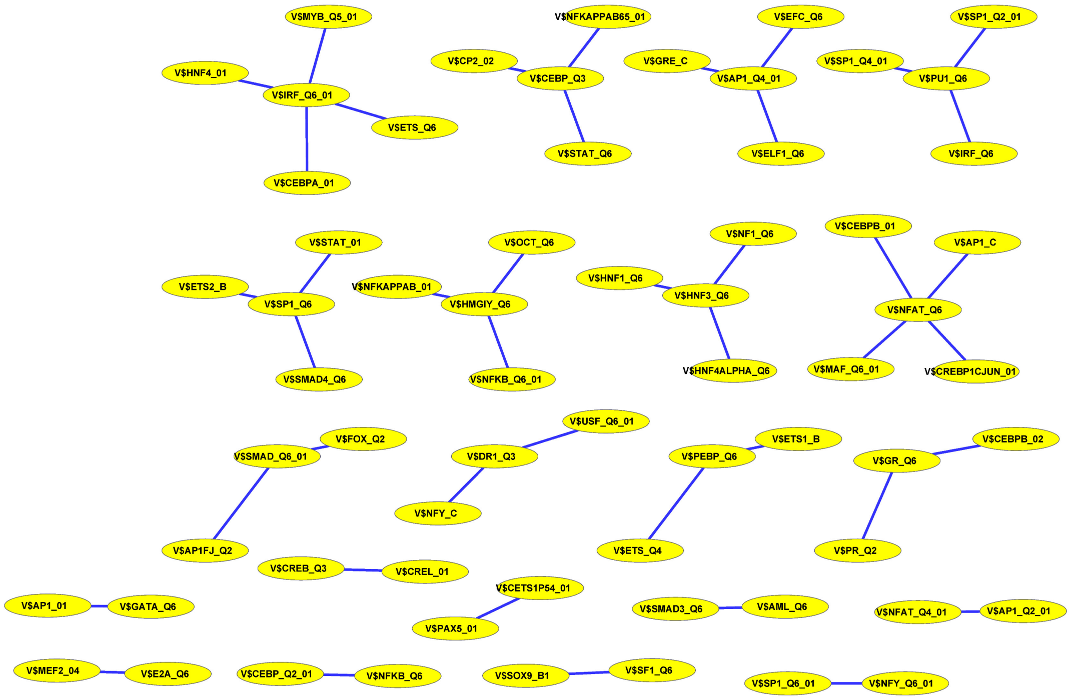


**Supplementary Figure 8.** Identified MatrixCatch pairs after utilization of the Markov clustering algorithm for day 13 - day 29 (early cardiac maturation stage).

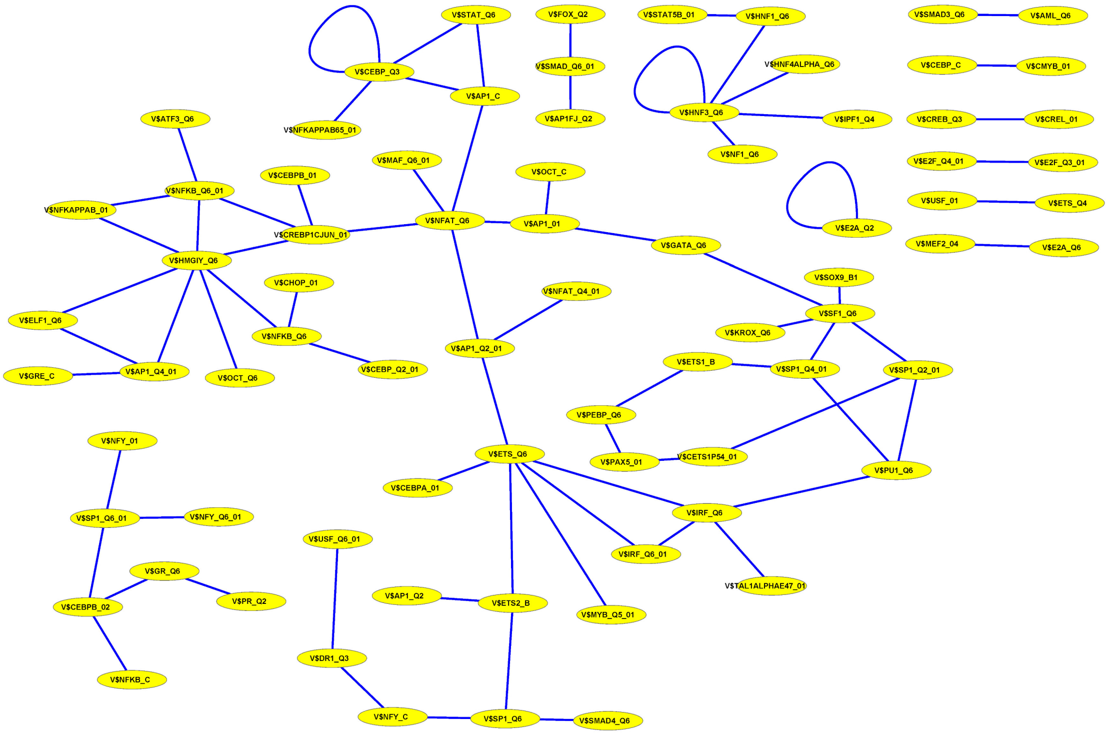


**Supplementary Figure 9.** Identified MatrixCatch pairs at day 29 - day 60 (late cardiac maturation stage).

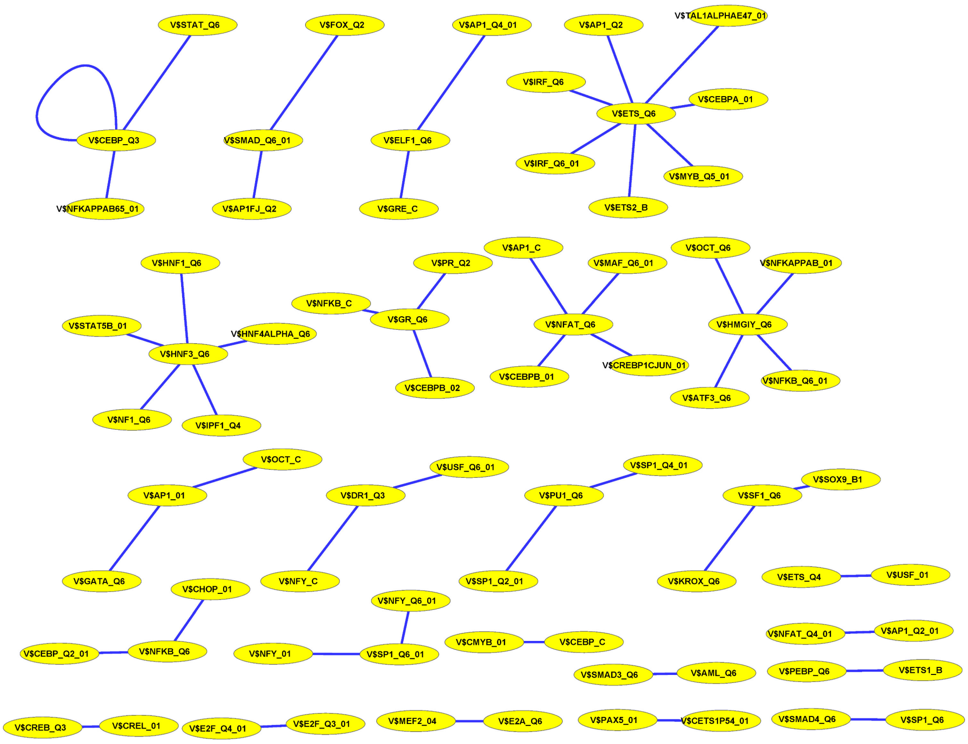


**Supplementary Figure 10.** Identified MatrixCatch pairs after utilization of the Markov clustering algorithm for day 29 - day 60 (late cardiac maturation stage).
